# Genome-wide association mapping and genomic prediction for CBSD resistance in *Manihot esculenta*

**DOI:** 10.1038/s41598-018-19696-1

**Published:** 2018-01-24

**Authors:** Siraj Ismail Kayondo, Dunia Pino Del Carpio, Roberto Lozano, Alfred Ozimati, Marnin Wolfe, Yona Baguma, Vernon Gracen, Samuel Offei, Morag Ferguson, Robert Kawuki, Jean-Luc Jannink

**Affiliations:** 1 National Crop Resources Research Institute, NaCRRI, P.O. Box, 7084 Kampala, Uganda; 2 West Africa Center for Crop Improvement, , (WACCI), University of Ghana, Accra, Ghana; 3 School of Integrative Plant Sciences, Section of Plant Breeding and Genetics, Cornell University, Ithaca, New York, USA; 4 US Department of Agriculture, Agricultural Research Service (USDA-ARS), Ithaca, New York, USA; 5 International Institute for Tropical Agriculture (IITA), Nairobi, Kenya

## Abstract

Cassava (*Manihot esculenta* Crantz) is an important security crop that faces severe yield loses due to cassava brown streak disease (CBSD). Motivated by the slow progress of conventional breeding, genetic improvement of cassava is undergoing rapid change due to the implementation of quantitative trait loci mapping, Genome-wide association mapping (GWAS), and genomic selection (GS). In this study, two breeding panels were genotyped for SNP markers using genotyping by sequencing and phenotyped for foliar and CBSD root symptoms at five locations in Uganda. Our GWAS study found two regions associated to CBSD, one on chromosome 4 which co-localizes with a *Manihot glaziovii* introgression segment and one on chromosome 11, which contains a cluster of nucleotide-binding site-leucine-rich repeat (*NBS-LRR*) genes. We evaluated the potential of GS to improve CBSD resistance by assessing the accuracy of seven prediction models. Predictive accuracy values varied between CBSD foliar severity traits at 3 months after planting (MAP) (0.27–0.32), 6 MAP (0.40–0.42) and root severity (0.31–0.42). For all traits, Random Forest and reproducing kernel Hilbert spaces regression showed the highest predictive accuracies. Our results provide an insight into the genetics of CBSD resistance to guide CBSD marker-assisted breeding and highlight the potential of GS to improve cassava breeding.

## Introduction

Cassava (*Manihot esculenta* Crantz) is a primary source of income and dietary calories for millions of people, and the high starch content of its storage roots is exploited in industry^1^. Although cassava is a resilient crop, its production in East Africa is often constrained by viral diseases including cassava brown streak virus disease (CBSD) which causes significant yield loses^2–4^. This disease is caused by two virus species of the genus *Ipomovirus*, family Potyviridae: *cassava brown streak virus* (CBSV) and *Ugandan cassava brown streak virus* (UCBSV)^5–7^. Both cassava brown streak viruses have successfully colonized a broad altitudinal range in South, East, and Central Africa and the steady spread of the disease is a threat to cassava production in West Africa^3,6^. In the field, CBSVs are transmitted by the whitefly (*Bemisia tabaci*) in a semi-persistent manner and through the exchange of infected cassava cuttings among farmers^8,9^. In susceptible clones, the viruses cause a myriad of symptoms including yellow chlorotic patterns along minor veins of leaves, necrotic streaks on the stems and brown or grey corky root necrosis^10–15^.

Breeding for durable CBSD resistance, through the development and propagation of CBSD-resistant varieties, has been the standard strategy to restrict disease spread. To date varieties with immunity to CBSVs from conventional breeding have not been reported. However, resistance in the form of restricted virus accumulation has been demonstrated, as has reduced symptom expression and recovery after clonal propagation^7,16,17^.

Numerous factors have hindered the rate of genetic progress in CBSD resistance breeding using conventional breeding approaches. These factors include the availability of suitable levels of resistance, the lack of well-characterized CBSD resistant varieties, genotype by environment interaction^18–20^, inconsistent year-to-year symptom expression, as well as reduced flowering, length of the breeding cycle, limited genetic diversity and slow rate of multiplication of planting materials^21,22^. Conventional cassava breeding can take three to six years from seedling germination to multi-location yield trials and additional years are required for evaluation of promising genotypes before superior clones are released as varieties^23^.

So far, few studies have used genomic-enabled approaches to increase the understanding of the mechanism of resistance and identify candidate genes and/or molecular markers associated with CBSD resistance or tolerance^19,20,24,25^. Recently, a transcriptome analysis of two cassava varieties, Namikonga (resistant) and Albert (susceptible), has facilitated the identification of a set of candidate genes that collectively may confer resistance to CBSD in Namikonga^25^. QTL mapping in biparental crosses successfully identified a set of QTL and candidate genes associated with resistance to CBSD induced root necrosis and CBSD foliar symptoms^19,20^. Notably, the characterization of QTL regions associated with CBSD resistance in the Tanzanian local cultivar Namikonga and the Tanzanian landrace Kiroba, suggest that some of those regions were introgressed from *Manihot glaziovii*^19,20^.

Crop biotechnology approaches such as RNAi technology have also been proposed to accelerate the integration of CBSD resistance into farmer-preferred cultivars^26–28^. Evaluation in the greenhouse and the field of transgenic lines, has shown efficient, durable and stable siRNA-derived resistance to CBSD across agro-ecological regions^28^.

Currently, genetic improvement of cassava is undergoing rapid change through the implementation of genomic-enabled tools by breeding programs in Africa (www.nextgencassava.org). Preliminary studies suggest that Genome-wide association studies (GWAS) and genomic selection (GS) are effective approaches for cassava breeding. Genome-wide association mapping studies in cassava have led to the identification of QTL regions associated with cassava mosaic disease resistance (CMD)^29^, beta-carotene content^30^ and dry matter content^31^. Genomic selection is particularly promising as an alternative method to marker-assisted selection (MAS) and conventional phenotypic selection because it can accelerate genetic gains due to the selection of parental genotypes with superior breeding values at the seedling stage based on genotypes alone^32–34^. Indeed, the evaluation of the performance of genomic prediction models using phenotypic and genotyping by sequencing (GBS) datasets from three African cassava breeding programs highlight the potential of GS as a breeding tool for some traits^23,35,36^.

In the present study, we followed a GWAS approach in combination with genomic prediction to unravel the genetic architecture of CBSD in two Ugandan breeding populations. The objectives of this study were: (1) to identify sources of resistance to CBSD in the GWAS panel, (2) to assess the current predictive accuracy for CBSD (3) to identify genomic prediction models that account for CBSD genetic architecture including the evaluation of a synergistic implementation of GWAS and GS and (4) to identify significant polymorphisms to guide CBSD marker-assisted breeding to improve cassava breeding in the face of increasing disease threats to agricultural production.

## Results

### Phenotypic variability

Foliar and root disease scoring was performed according to a standard CBSD scoring scale that ranges from 1 to 5 (Supplementary Fig. S1). The distribution of CBSD de-regressed BLUPs is presented in Supplementary Figs S2 and S3. Both GWAS panels exhibited differential response foliar symptom response to CBSVs at 3, 6 and 9 months after planting (MAP) and root severity (CBSDRS) at 12 MAP as demonstrated by the variability of the de-regressed BLUPs. Interestingly, clones which displayed an intermediate response were more abundant than clones with a susceptible or resistance response.

Phenotypic correlations were calculated within panels and within and across locations for foliar symptom severity scores at 3 MAP and 6 MAP and CBSDRS. Correlations across locations for each panel are given in Supplementary Figure S4 and Supplementary Tables S2 and S3. Clear differences were observed in CBSD severity scores. For Panel 1, the lowest correlation value corresponded to CBSDRS and was found between the locations Ngetta and Kasese (0.09) while the highest correlation value, which also corresponded to CBSDRS, was found between the locations Namulonge and Kasese (0.60) (Supplementary Table S2A). For Panel 2, correlation values ranged across locations between −0.08 at 9 MAP (Namulonge-Kamuli) and 0.51 at 3 MAP (Kamuli-Serere) (Supplementary Table S2B).

Across traits within locations, the highest correlation values were found in Panel 1 for foliar scorings 3 MAP and 6 MAP (r^2^ > 0.5) (Supplementary Table S3A). For Panel 2, correlation across traits varied depending on the location. Nonetheless, correlations across foliar traits were higher than those found between foliar and root symptom severity scores (Supplementary Table S3B).

### Broad-sense and SNP heritability

For both panels and across locations the heritability estimates for CBSD symptom severity scorings at 3 MAP, 6 MAP, 9 MAP, and CBDRS were low to intermediate. Broad-sense heritability (H^2^) estimates across panels and locations spanned a wide range of values from 0.11 for 3 MAP at Namulonge (hotspot CBSD location) (Panel 1) to 0.75 for scorings 9 MAP at Kamuli (moderate CBSD prevalence) (Panel 2) (Table 1). Specifically, for GWAS Panel 1, broad-sense heritability (H^2^) estimates ranged between 0.11 for 3 MAP at Namulonge and 0.73 for CBSDRS at Ngetta (low CBSD pressure). For GWAS Panel 2, H^2^ estimates ranged from 0.24 for scorings 3 MAP to 0.75 at Kamuli for 9 MAP.

**Table 1:** Broad-sense heritability (H^2^) and SNP heritability (h^2^) of foliar and root CBSD severity. For each panel, the heritability values were estimated per location and by combining locations (see methods).

Trait	H^2^	h^2^	LOCATION-YEAR	Panel
CBSD 3 MAP	0.11	0.32	NAMULONGE	1
CBSD 6 MAP	0.31	0.39	NAMULONGE	1
CBSDRS	0.55	0.59	NAMULONGE	1
CBSD 3 MAP	0.43	0.48	NGETTA	1
CBSD 6 MAP	0.51	0.53	NGETTA	1
CBSDRS	0.73	0.72	NGETTA	1
CBSD 3 MAP	0.27	0.29	KASESE	1
CBSD 6 MAP	0.21	0.27	KASESE	1
CBSDRS	0.39	0.47	KASESE	1
CBSD 3 MAP	0.61	0.17	MULTI-LOCATION	1
CBSD 6 MAP	0.35	0.31	MULTI-LOCATION	1
CBSDRS	0.37	0.34	MULTI-LOCATION	1
CBSD 3 MAP	0.60	0.37	NAMULONGE	2
CBSD 6 MAP	0.60	0.32	NAMULONGE	2
CBSD 9 MAP	0.68	0.34	NAMULONGE	2
CBSDRS	0.24	0.53	NAMULONGE	2
CBSD 3 MAP	0.63	0.28	SERERE	2
CBSD 6 MAP	0.60	0.28	SERERE	2
CBSD 9 MAP	0.73	0.34	SERERE	2
CBSDRS	0.15	0.48	SERERE	2
CBSD 3 MAP	0.56	0.27	KAMULI	2
CBSD 6 MAP	0.62	0.29	KAMULI	2
CBSD 9 MAP	0.75	0.34	KAMULI	2
CBSDRS	0.28	0.44	KAMULI	2
CBSD 3 MAP	0.42	0.28	MULTI-LOCATION	2
CBSD 6 MAP	0.47	0.34	MULTI-LOCATION	2
CBSD 9 MAP	0.56	0.38	MULTI-LOCATION	2
CBSDRS	0.25	0.33	MULTI-LOCATION	2

CBSD: Cassava brown streak disease, MAP: months after planting, CBSDRS cassava brown streak root severity.

Further, we combined genotypic and phenotypic data and estimated SNP heritability values using variance components that were obtained as a result of fitting a one-step model for each panel, each location, and multi-location datasets. For Panel 1, H^2^ and SNP heritability values were approximately the same across locations except for Namulonge (3 MAP) that displayed an H^2^ value of 0.11 and for the multi-location dataset (3 MAP), which showed the most significant difference between H^2^ and SNP heritability estimates. For Panel 2, H^2^ estimates were consistently higher than SNP heritability estimates except for Namulonge, Serere, Kamuli and the multi-location dataset for CBSDRS.

### Genome-wide association study

**Figure 1:**
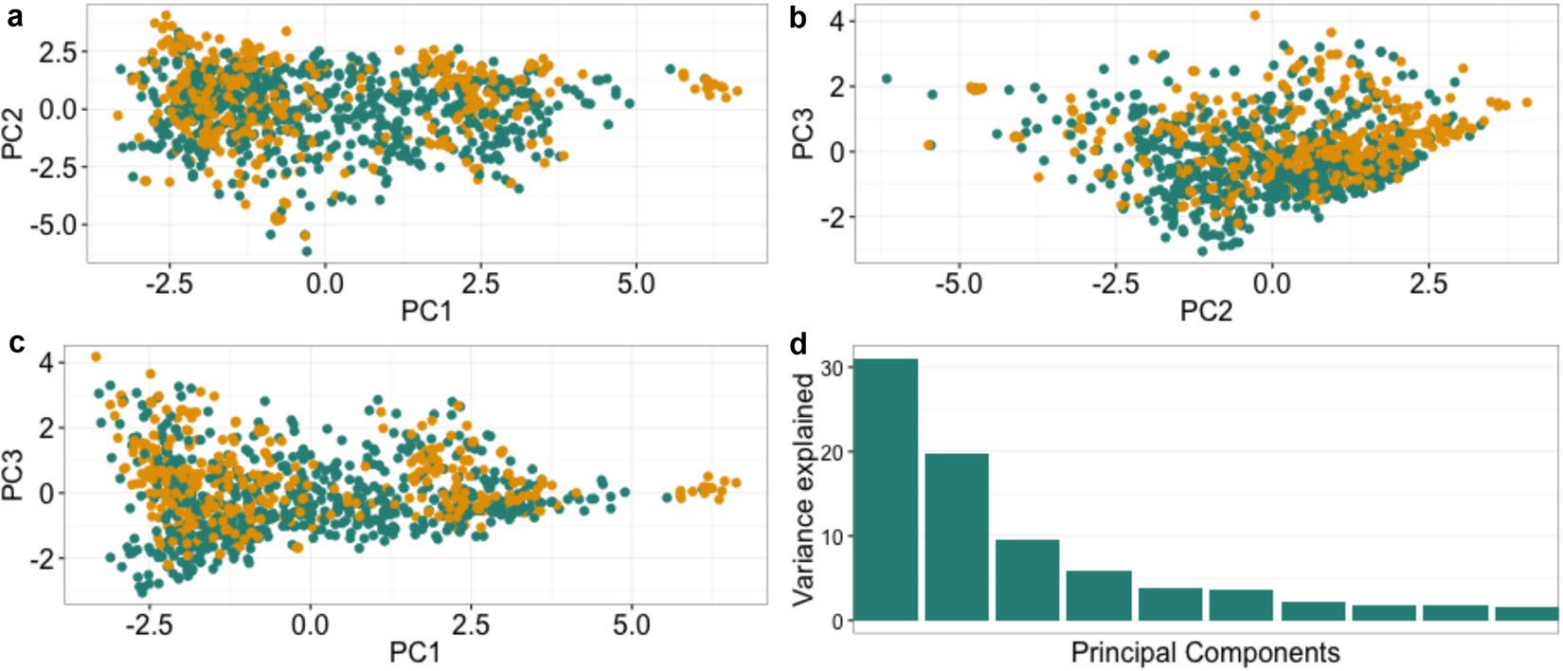
Plot of the first three principal components (PCs). Panel 1 and Panel 2 clones were used in the PC analysis. The top panels and the lower left panel display the distribution of clones in PC1-PC3. The lower right panel shows the variance explained by the first ten principal components. Colours correspond to members of Panel 1 (green) and Panel 2 (orange) clones.

The genetic structure of the GWAS panels was estimated by using principal components analysis (PCA). Overall, the first three principal components (PCs) accounted for 60% of the genetic variation observed in the data (Fig. 1), the first PC accounted for 30% of the observed variation while the second and third PCs contributed 20% and 10% respectively. Most clones showed no clear separation, which indicates the presence of only one cluster and low stratification across panels.

Manhattan plots of the genotype-phenotype associations at 3 MAP, 6 MAP, and CBDRS based on the combination of ~1000 clone multi-location de-regressed BLUPs are presented in Fig. 2. Additional GWAS analyses performed on each panel and location are given in Supplementary Tables S4 and S5 and Supplementary Figs S5–S12.

**Figure 2:**
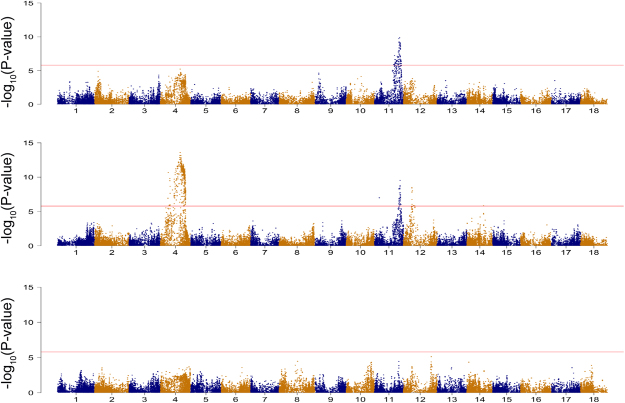
Manhattan plots of three CBSD severity scorings in leaves and roots. The GWAS results presented correspond to the combined dataset. Association tests were performed for CBSD symptom severity on leaves at (**a**) 3 and (**b**) 6 month after planting (MAP) and (**c**) on roots (CBSDRS). The horizontal line indicates the genome-wide significance level (−log_10_(P-value) = 5.9).

SNP markers with a −log_10_(P-value) which exceeded the Bonferroni threshold >5.9 were considered to be statistically significant and were further annotated into coding regions (genes) of the cassava genome (Supplementary Table S4). Using the multi-location dataset, 83 significant SNP markers were identified as being associated with foliar symptoms at 3 MAP. All the significant markers were located on chromosome 11 and from these, 61 were annotated within genic regions (Supplementary Table S4). The top SNP −log_10_(P-value) = 9.38 within the QTL on chromosome 11 explained 6% of the observed phenotypic variance (Supplementary Table S5). For foliar severity scores at 6 MAP, we identified SNP associations on chromosome 11, chromosome 4 and chromosome 12. On chromosome 11, 33 SNPs passed the Bonferroni threshold, and from these markers, 27 were annotated within genic regions. This QTL was in the same chromosomal location as that found for 3 MAP foliar CBSD QTL and explained 5% of the observed phenotypic variance (Fig. 2). Although several of the SNPs on chromosome 11 associated with 6 MAP CBSD exceeded the Bonferroni threshold, only six SNPs were in linkage disequilibrium (r^2^ > 0.6) with the highest −log_10_(P-value) SNP hit.

**Figure 3:**
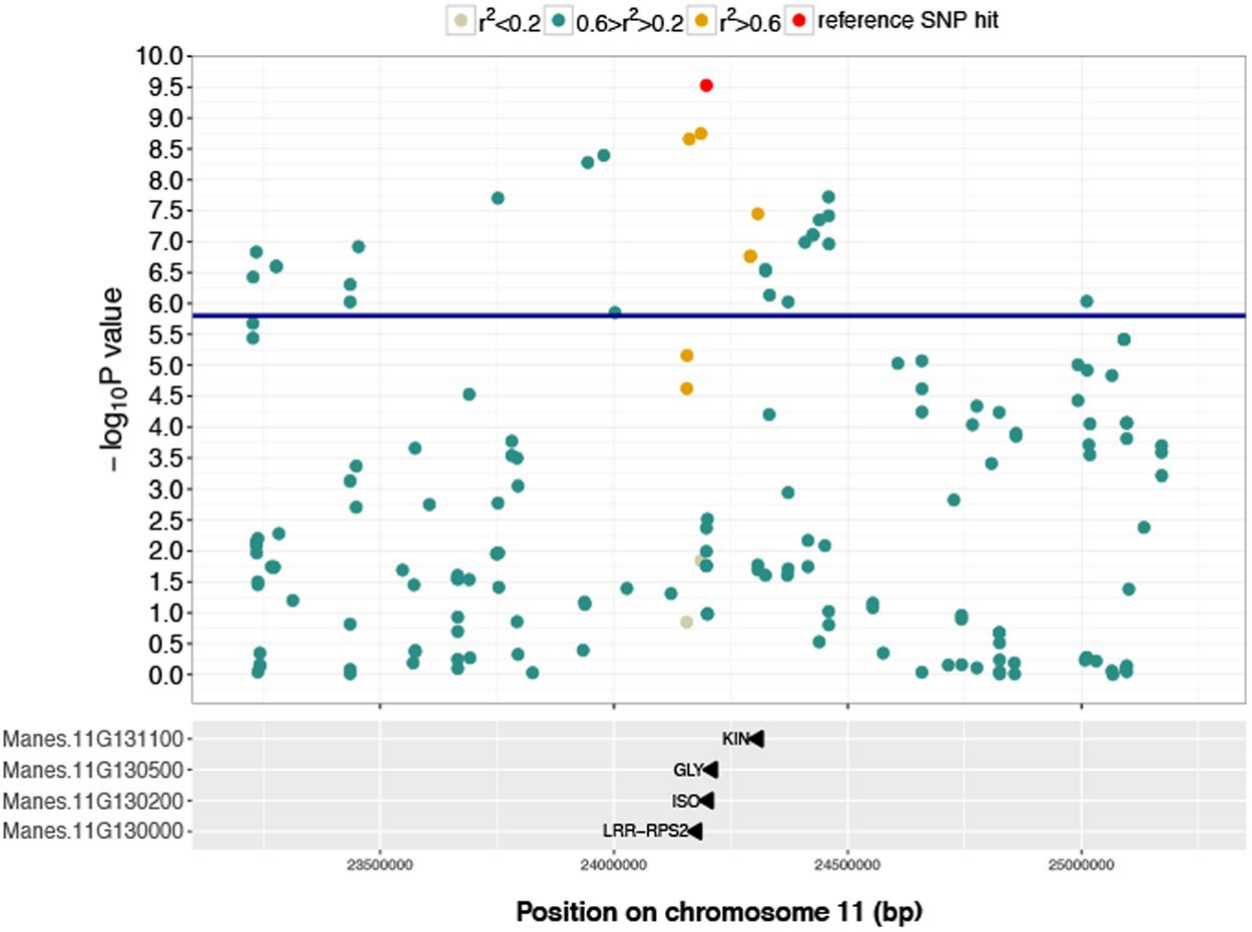
Local Manhattan plot surrounding the peak on chromosome 11. The plot spans a 2 Mb region on chromosome 11. At the top, the SNP indicated in red is the SNP with the highest −log_10_(P-value) = 9.38 on that chromosome for CBSD symptom severity at 3 MAP. Colours indicate the Pearson’s correlation coefficient (*r*^2^) between the top significant GWAS SNP hit on this chromosome and neighboring markers in the given window. Markers with *r*^*2*^ > 0.2 in annotated genes are indicated in the panel below.

Genes on chromosome 11 containing SNP markers, with a correlation value of r^2^ > 0.2 with the highest −log_10_(P-value) marker, were annotated and classified as candidate genes. The genes identified were Manes.11G130500, Manes.11G130000, Manes.11G130200 and Manes.11G131100. Manes.11G130500 is known to encode glycine-rich protein. Manes.11G130000 encodes a Leucine-rich repeat (LRR) containing protein. Manes.11G130200 encodes the trigger factor chaperone and *peptidyl-prolyl* trans. Finally, Manes.11G131100 encodes a protein kinase (Fig. 3).

**Figure 4:**
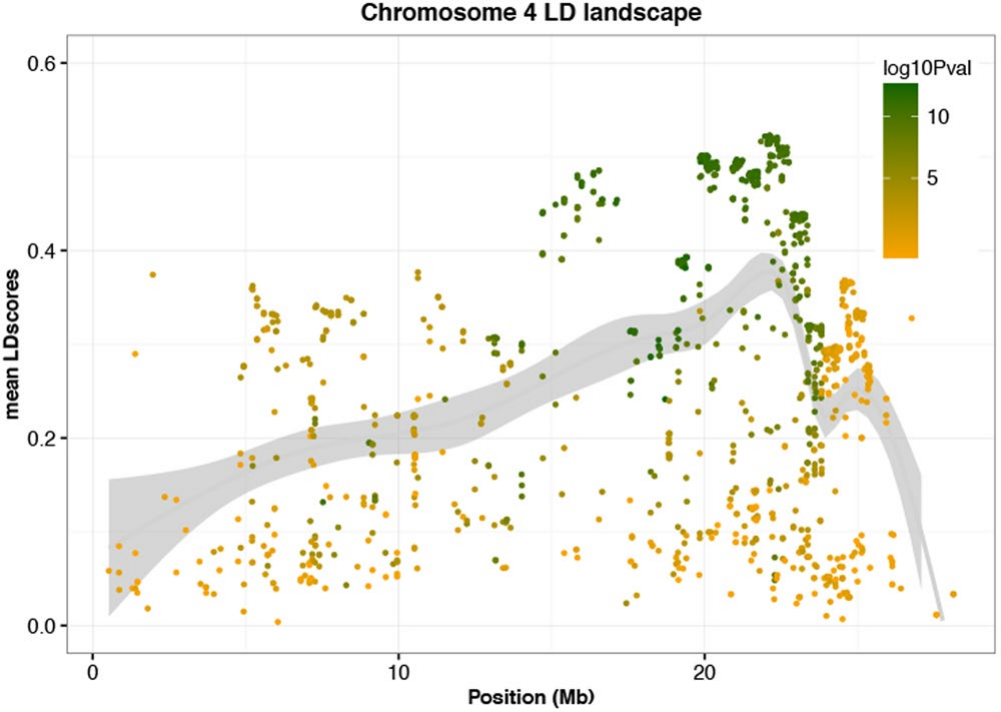
Local Manhattan plot of Chromosome 4. LD score values (*r*^2^) for each marker on chromosome 4 plotted against physical distances between markers. The smooth line represents a relative measure of the local LD in chromosome 4. Dot colors depend on the −log_10_(P-value) obtained for CBSD symptom severity at 6 MAP.

On chromosome 4 the significant SNPs defining the QTL were in high linkage disequilibrium (Fig. 4) and co-localized with an introgression segment from a wild relative of cassava (*M. glaziovii*)^37,38^. We further confirmed the presence and segregation of the introgressed genome segment in both panels using a set of diagnostic markers from *M. glaziovii* (Supplementary Fig. S13). Because of the high level of linkage disequilibrium at the QTL location, we do not highlight a single locus or loci as candidate gene(s) associated with CBSD foliar severity.

The significant QTL on chromosome 12 associated with foliar severity at 6 MAP had previously been found to be associated with CMD resistance in cassava^29^. To confirm the association of that QTL with foliar severity scores at 6 MAP we re-fit the first-step mixed-models used to obtain de-regressed BLUPs, this time including CMD severity as a fixed-effect covariate. After the corrected de-regressed BLUPs were included in the GWAS analysis, the QTL on chromosome 12 was no longer significant, and only the QTL on chromosomes 4 and 11 remained (Supplementary Fig. S14).

SNPs surpassing the Bonferroni threshold could not be identified for CBSDRS across panels. However, analysis of the multi-location data for Panel 1 identified significant regions of CBSDRS association on chromosomes 5, 11 and 18 (−log_10_(P-value) > 6.5), which explained 8, 6 and 10% of the phenotypic variance respectively.

### Genome-wide prediction

**Figure 5:**
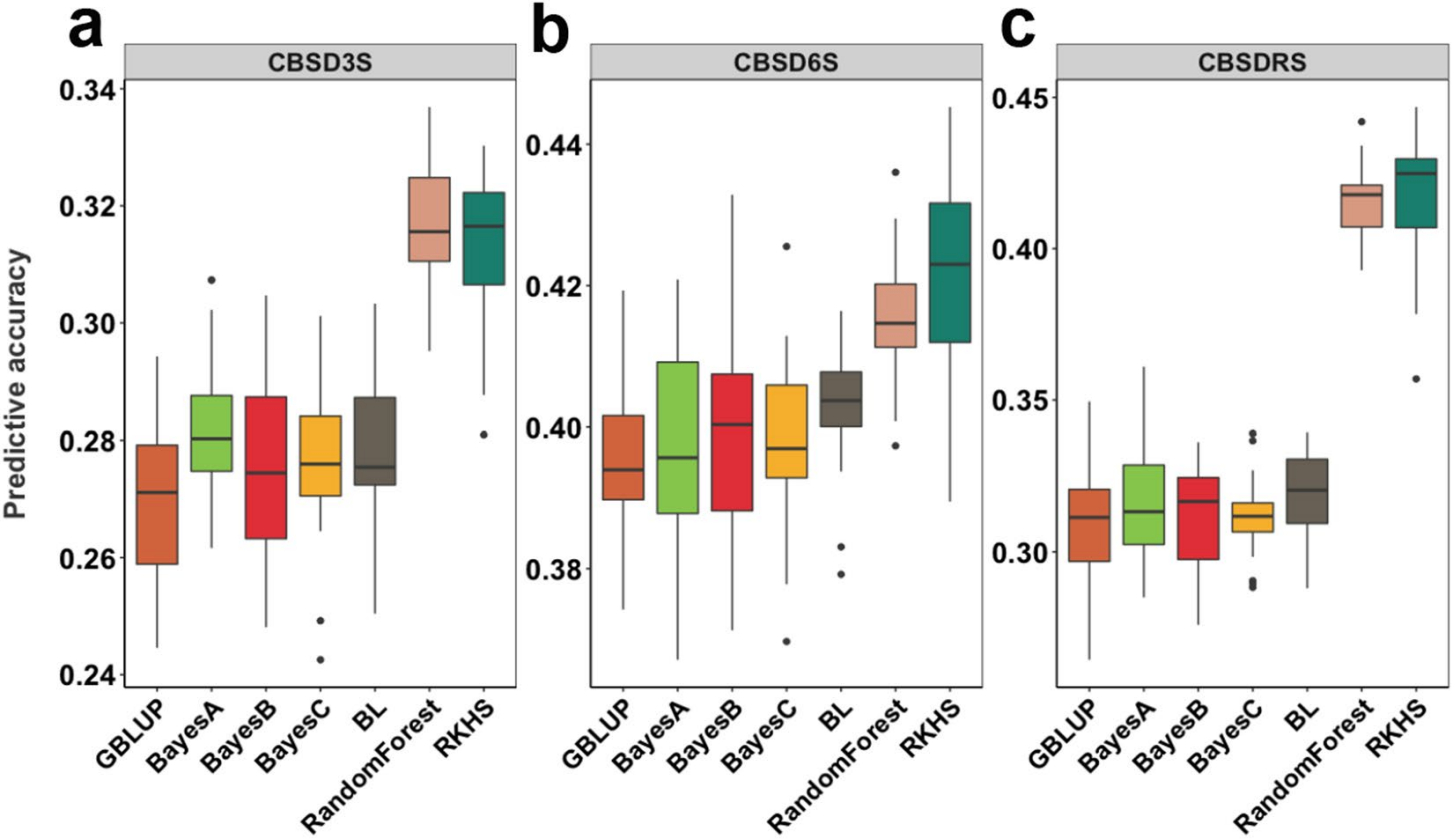
Plot of cross-generation prediction accuracies for CBSD severity symptoms. Seven genomic prediction methods (colors, x-axis within panels) were tested for CBSD symptom severity predictive accuracy (y-axis within panels) on leaves at (**a**) 3 and (**b**) 6 month after planting (MAP) and on roots (**c**) 12 MAP.

Using the combined dataset, we compared the performance of seven genomic prediction models with different assumptions on trait genetic architecture. Some model predictions represent genomic estimated breeding values (GEBV) in that they are sums of additive effects of markers, while other model predictions represent genomic estimated total genetic values (GETGV) because they include non-additive effects. Prediction accuracy for CBSD related traits had mean values across methods of 0.29 (3 MAP), 0.40 (6 MAP) and 0.34 (CBSDRS) (Fig. 5 and Supplementary Table S6A).

These accuracies varied from 0.27 (BayesB and GBLUP) to 0.32 (RF) for 3 MAP, from 0.40 (most methods) to 0.41 (RF) and 0.42 (RKHS) for 6 MAP and from 0.31 (BayesA, B, C and GBLUP) to 0.42 (RF and RKHS) for CBSDRS. It is clear from the results that higher predictive accuracies were consistently achieved when using RF and RKHS for the prediction of both foliar and root CBSD resistance traits. For foliar symptoms, the increase in predictive accuracy using RF and RKHS was modest in comparison to GBLUP, whereas for CBSDRS the predictive accuracy increased from 0.31 using GBLUP to 0.42 using RF and RKHS models.

### GWAS-guided genomic prediction

Based on the genome-wide association results, we identified for foliar 3 MAP and 6 MAP and CBSDRS the strongest marker associations on chromosomes 4 and 11. To test if GWAS results can help to improve genomic prediction accuracy markers from chromosomes 4, 11 and markers on other chromosomes were used independently to construct covariance matrices that were fitted together in a multikernel GBLUP model (Supplementary Fig. S15). For all CBSD traits, the mean predictive accuracy results from the single-kernel GBLUP model were similar to the mean total predictive accuracy following the multi-kernel approach (Supplementary Table S6B). However, the contribution of the individual kernels to the total predictive accuracies was different. For example, in the multikernel GBLUP model for CBSD 3 MAP (0.27), the highest contribution to the total predictive accuracy came from chromosome 11 and the rest of the genome (0.19). In contrast, the multikernel GBLUP model for 6 MAP gave the highest predictive accuracy (0.40) with the majority of prediction coming from chromosome 4 (0.29). Finally, the multikernel GBLUP approach for CBSDRS had a total predictive accuracy of 0.30 with the rest of the genome (0.29) contributing most to the total predictive accuracy (Supplementary Fig. S15).

## Discussion

Efforts to understand CBSD resistance have focused on population development and QTL mapping, viral strain characterization, development of transgenic lines and evaluation of local and elite cassava genotypes to identify possible sources of resistance^10,19,20,39,40^. Although QTL and candidate genes associated with resistance to CBSD have been identified through bi-parental mapping, further transcriptional studies are needed to validate results and uncover the genetics of resistance to CBSD^19,20,25^.

We evaluated two GWAS panels from the NaCRRI breeding program in Uganda for CBSD severity symptoms in leaves and roots at locations with different CBSD disease pressure and environmental conditions. In both GWAS panels, the frequency distribution of scores within locations and the range of correlation values across locations (−0.08 to 0.60) reflect the differences in CBSD pressure at these locations. For example, Kasese and Namulonge are both hotspots for CBSD and showed a higher correlation in comparison to Kasese and Ngetta, which differ on their CBSD prevalence. The high variability observed within and across GWAS panels reflects differences in population composition, trait genetic architecture, field design and environmental effects including differences in virus strains or species which may contribute to poor correlations across locations.

In addition to scoring variability within and across locations, both panels showed variation in foliar CBSD symptom expression at 3, 6 and 9 MAP and root necrosis CBSDRS. These results are consistent with the observation that symptom expression changed with the age of the plant, with the loss of lower symptomatic leaves or symptoms being obscured in older plants^16,41^. Nonetheless, the correlation among foliar CBSD severities, in both panels and across locations, were consistently higher than the correlation between foliar and root severities. Similar to other studies^20,21^, our results demonstrate variability of symptom expression across environments and suggest that mechanisms of CBSD resistance operate somewhat independently in leaves and storage roots^17^, or are under different genetic control.

Given the availability of multi-location phenotypic data and GBS genotyping datasets, we assessed the potential of GWAS to identify QTL associated with CBSD resistance in leaves and storage roots. For all traits, several factors played a role in the identification of significantly associated SNPs including differences in panel size, age of the plant at scoring, CBSD prevalence and environmental condition at the different locations. Based on preliminary GWAS results in individual panels, we decided to increase the study power and resolution of our GWAS by combining multi-location scores from both panels. GWAS in the combined population detected SNPs significantly associated (P-value > 5.9) with foliar CBSD symptoms at 3 and 6 MAP on chromosomes 4 and 11. In contrast, no significant SNPs associated with CBSDRS could be detected, possibly due genotype-by-environment (GxE) interaction with CBSD and polygenic control or mechanisms of resistance under different genetic control contributing to CBSDRS^19^. However, when Panel 1 was analyzed independently, significant regions associated with CBSDRS were identified on chromosomes 5, 11 and 18. Similarly, Nzuki *et al*.^19^ in an analysis of a biparental population identified a region on chromosome 5 associated with root necrosis and a region on chromosome 11 associated with both root necrosis and foliar symptoms. Moreover, Masumba *et al*.^20^ found chromosomes 11 and 18 associated with CBSD root necrosis. Both QTL mapping studies were developed from crosses between Tanzanian clones and support the idea that much of the CBSD resistance present in the Ugandan breeding population has its origin in Tanzania.

Genomic annotation of the foliar GWAS results showed that a cluster of genes underlies the significant QTL associated with foliar disease resistance on chromosome 11; candidate genes that were identified for further study are Manes.11G131100, Manes.11G130500, Manes.11G130200 and Manes.11G130000. Lozano *et al*.^42^ previously reported Manes.11G130000 when studying the distribution of NBS-LRR gene family in the cassava genome. In addition,Manes.11G130000 was among the differentially expressed genes during early transcriptome response to brown streak virus infection in the susceptible line 60444 from the ETH cassava germplasm collection^40^. The QTL on chromosome 11 is particularly unstable across locations, which may be related to NBS-LRR genes conferring resistance to a particular viral strain^3,7^.

Throughout the 1940s and 1950s at the Amani Research Station in Tanzania, *M. glaziovii* and cassava varieties of Brazilian origin were successfully used for crosses to obtain varieties which showed high levels of field resistance to CBSD^43^. Similar to Bredeson *et al*.^38^, in our study, we identified widespread evidence for interspecific hybridization in the Ugandan cassava breeding panels. One of these introgression regions is located on chromosome 4, which in our study was associated with foliar severity. However, the degree of linkage disequilibrium in that region of chromosome 4 was a major constraint for the identification of a gene or genes responsible for CBSD resistance^38^. Interestingly, this region was not detected in the QTL mapping population involving the inter-specific hybrid, Namikonga^21^, but was detected in a cross with Kiroba^20^, another inter-specific hybrid presumably originating from the Amani breeding program.

Genomic selection (GS) can accelerate genetic gains through the use of phenotypic and genotypic data from a training population for early selection of seedlings to develop superior varieties^32–34^. We applied genomic prediction models that have different underlying assumptions: the GBLUP model assumes an infinitesimal genetic architecture, Bayesian methods such as BayesA and BayesB relax the assumption of common variance across marker effects^44–46^ and RKHS and RF can model epistasis. For all traits, using the combined dataset, we found moderate predictive accuracies in the 0.27–0.42 range; in general, predictive accuracies were in accordance with broad-sense heritability estimates, which ranged for Panel 1 between 0.37–0.61 and Panel 2 between 0.25–0.46. In general, we observed a superiority of the RF and RKHS models to predict both CBSD foliar symptoms and root necrosis, though the increase in predictive accuracy was more prominent for root necrosis. Similar to our results, previous studies that contrasted the performance of various prediction methods in cassava showed that RF and RKHS displayed higher predictive accuracy, particularly with phenotypes known to have a significant amount of non-additive genetic variation such as yield-related traits^23^. Based our findings, non-additive effects are likely to play a role shaping CBSD resistance in cassava in particular for root necrosis.

Even though *a priori* knowledge of the loci associated with a trait is not needed for GS, we tested a multiple kernel approach by fitting three kernels with genomic relationship matrices constructed with SNP markers from chromosomes 4 (G_chr4_), 11 (G_chr11_) and SNPs from other chromosomes (G_allchr-[4,11]_). While the use of a multiple kernel approach did not increase predictive accuracies, the highest contribution of each kernel to the total predictive accuracy modeled the genetic architecture of CBSD traits. Based on our results, we conclude that genomic selection is a promising breeding tool for selecting for CBSD resistance. Based on our results, we conclude that genomic selection is a promising breeding tool for selecting for CBSD resistance and that the use of prediction models that consider both additive and non-additive effects could be advantageous.

Cassava brown streak disease is devastating cassava production in regions already affected by CBSVs and poses a high risk to countries in Central and West Africa where CBSD is not currently present. The GWAS and GS results presented in this study support previous findings which indicate that resistance to CBSD is polygenic^39^ and unstable across environments, which is an indication of quantitative resistance^47^. Although we were able to identify a candidate NBS-LRR gene on chromosome 11, the function of this gene in CBSD resistance requires further validation and more importantly, there is the possibility that this gene might not be a source of durable resistance to CBSVs. Further work will require screening large diversity panels, possibly including wild relatives, in multiple environments, and efforts to identify QTL specific to certain viral strains to further support the development of CBSD resistance.

## Materials and Methods

### Plant material and phenotyping

We assembled two cassava GWAS panels composed of 429 and 872 clones, respectively. These clones represented germplasm diversity derived from the International Institute for Tropical Agriculture (IITA) and the International Center for Tropical Agriculture (CIAT) (Supplementary Table 1).

GWAS Panel 1 clones were evaluated in replicated two-row 5 m plots in an alpha lattice field design from 2012 through 2014 at Namulonge, Ngetta, and Kasese in Uganda. While GWAS Panel 2 clones were evaluated in single row 10 m plots in an augmented design^48,49^ with 6 checks randomized in each incomplete block of 25 clones during the 2014 to 2015 season at Namulonge, Kamuli, and Serere.

Foliar disease severity was visually scored following the standard field phenotyping protocol for CBSD at 3, 6, and 9 months after planting (MAP) on a 1–5 scale^13,50,51^. Additional method description can be found in the supplementary methods section and Supplementary Fig. S1.

### Two-stage genomic analyses

The first stage in data analysis involved accounting for trial design using a linear mixed model to obtained de-regressed BLUPs, and the second stage involved the use of de-regressed BLUPs in GWAS and Genomic prediction.

For Panel 1 we fit the model:

1 $$y={\bf{X}}\beta +{{\bf{Z}}}_{{\bf{c}}{\bf{l}}{\bf{o}}{\bf{n}}{\bf{e}}}c+{{\bf{Z}}}_{{\bf{b}}{\bf{l}}{\bf{o}}{\bf{c}}{\bf{k}}}b+\varepsilon $$

In this model, ***β*** included a fixed effect for the population mean and location. The incidence matrix **Z**_**clone**_ and the vector *c* represent a random effect for clone $c \sim {\rm{N}}(0,{\bf{I}}{\sigma }_{c}^{2})$ and ${\bf{I}}$ represents the identity matrix. The range variable, which is the row or column along which plots are arrayed, is nested in location-rep and is represented by the incidence matrix **Z**_**range(loc.)**_ and random effects vector $r \sim {\rm{N}}(0,{\bf{I}}{\sigma }_{r}^{2})$. Block effects were nested in ranges and incorporated as random effects with incidence matrix Z_block(range)_ and effects vector $b \sim {\rm{N}}(0,{\bf{I}}{\sigma }_{b}^{2})$. Residuals *ε* were distributed $\varepsilon  \sim {\rm{N}}(0,{\bf{I}}{\sigma }_{\varepsilon }^{2})$.

For Panel 2 we fit the model

2 $${\boldsymbol{y}}=\,{\bf{X}}\beta +{{\bf{Z}}}_{{\bf{c}}{\bf{l}}{\bf{o}}{\bf{n}}{\bf{e}}}c\,+{{\bf{Z}}}_{{\bf{b}}{\bf{l}}{\bf{o}}{\bf{c}}{\bf{k}}}b+\varepsilon $$

Where *y* was the vector of raw phenotypes, ***β*** included fixed effects for the population mean, the location and finally an effect for checks. The incidence matrix **Z**_**clone**_ and the vector *c* are the same as the model above, and the blocks were also modeled with incidence matrix ${{\bf{Z}}}_{{\bf{b}}{\bf{l}}{\bf{o}}{\bf{c}}{\bf{k}}}$ and **b** represents the random effect for block. The best linear predictors (BLUPs) of the clone effect (*ĉ*) were extracted as de-regressed BLUPS following the formula^52^:

3 $${\bf{d}}{\bf{e}}{\bf{r}}{\bf{e}}{\bf{g}}{\bf{r}}{\bf{e}}{\bf{s}}{\bf{s}}{\bf{e}}{\bf{d}}\,{\bf{B}}{\bf{L}}{\bf{U}}{\bf{P}}=\frac{{\bf{B}}{\bf{L}}{\bf{U}}{\bf{P}}}{1-\frac{{\bf{P}}{\bf{E}}{\bf{V}}\,}{{{\boldsymbol{\sigma }}}_{{\bf{c}}}^{2}}}$$

Where PEV is the prediction error variance of the BLUP and ${{\boldsymbol{\sigma }}}_{{\bf{c}}}^{2}$ is the clonal variance component. We used the *lmer* function from the *lme4* R package^53^ to fit the models described above.

### Genotyping by sequencing (GBS)

Total genomic DNA was extracted from young leaves according to standard procedures using the DNAeasy plant mini extraction kit^54^. GBS libraries were constructed using the ApeKI restriction enzyme as previously described^55^. Marker genotypes were called using TASSEL GBS pipeline V4^56^ after aligning the reads to the Cassava v6 reference genome^57,58^. Markers with more than 60% missing calls were removed.

The resulting marker dataset consisted of 173,647 bi-allelic SNP markers and was imputed using Beagle 4.1^59^. After imputation, 63,016 SNPs had an AR^2^ (Estimated Allelic r-squared) higher than 0.3 and were kept for downstream analysis. From the remaining imputed markers, 41,530 had a minor allele frequency (MAF) higher than 0.01.

### Genetic correlations and heritability estimates

Correlation across CBSD traits and within each location was estimated using using the de-regressed BLUP values obtained after fitting the aforementioned linear mixed model. Broad-sense heritability values on an entry-mean basis were calculated using the variance components estimated using the mixed-models described above, which represent the first-step of the two-step genomic analysis.

Finally, SNP-based heritability was calculated for each GWAS panel by fitting a single-step mixed-effects model using the *emmreml* function from the *EMMREML* R package^60^. The random effect was modeled as having a co-variance proportional to the kinship matrix, which was calculated using the *A.mat* function from the *rrBLUP* R package^61^.

### Genome-wide association analysis for CBSV severity

A principal component analysis (PCA) was performed across panels to identify any population stratification between the two GWAS panels. We used the imputed dataset of 63,016 SNP markers to calculate the PCs with the function *princomp* in R^62^.

With the imputed dataset of 63,016 SNP markers and 986 individuals, GWAS was performed using a mixed linear model association analysis (MLMA) accounting for kinship and filtering by MAF > 0.05 as implemented in GCTA (v 1.26.0)^63^. We followed a leave one chromosome out approach in which the chromosome with the tested candidate SNP markers is excluded from the genomic relationship (GRM) calculation. Manhattan plots were generated using R package *qqman* with a Bonferroni threshold of 5.9^64^.

### Genomic prediction models

#### GBLUP

In this prediction model, the GEBVs are obtained after fitting a linear mixed model where the genomic realized relationship matrix is based on SNP marker dosages. Accordingly, the genomic relationship matrix was constructed using the function *A.mat* in the R package rrBLUP^61^. GBLUP predictions were made with the function *emmreml* in the R package EMMREML^60^.

#### Multi-kernel GBLUP

Because the most significant QTLs for foliar CBSD severity at 3 and 6 MAP were mapped on chromosomes 4 and 11 (this paper) we followed a multi-kernel approach by fitting three kernels with genomic relationship matrices constructed with SNP markers from chromosomes 4 (G_chr4_), 11 (G_chr11_) and SNPs from the other chromosomes (G_allchr-[4,11]_). Multi-kernel GBLUP predictions were made with the function *emmremlMultiKernel* in the R package EMMREML^60^.

#### Reproducing kernel Hilbert spaces (RKHS)

We use a Gaussian kernel function:

4 $${K}_{ij}=\exp (-({{\rm{d}}}_{{\rm{ij}}}{\rm{\theta }}))$$

where *K*_*ij*_ is the measured relationship between two individuals, *d*_*ij*_ is their Euclidean genetic distance based on marker dosages, and θ is a tuning (“bandwidth”) parameter that determines the rate of decay of correlation among individuals. This function is nonlinear, and the kernels used for RKHS can capture non-additive as well as additive genetic variation^65^. To fit a multiple-kernel model with six covariance matrices, we used the *emmremlMultiKernel* function in the EMMREML package, with the following bandwidth parameters: 0.0000005, 0.00005, 0.0005, 0.005, 0.01, 0.05 (Multi-kernel RKHS) and allowed REML to find optimal weights for each kernel.

#### Bayesian maker regressions

We tested four Bayesian prediction models: BayesCpi^46^, the Bayesian LASSO^66^, BayesA, and BayesB^32^. The Bayesian models we tested allow for alternative genetic architecture by way of differential shrinkage of marker effects. We performed Bayesian predictions with the R package BGLR^67^.

#### Random Forest

Random Forest (RF) is a machine learning method used for regression and classification^68–70^. Random Forest regression with marker data has been shown to capture epistatic effects and has been successfully used for prediction^70–74^. We implemented RF using the Random Forest package in R^75^ with the parameter*, ntree* set to 500 and the number of variables sampled at each split (*mtry*) equal to 300.

### Introgression Segment Detection

To identify introgression segments in the two GWAS panels, we followed the approach described in Bredeson *et al*.^38^. We used the *M. glaziovii* diagnostic markers identified in Supplementary Dataset 2 of Bredeson *et al*.^38^. These ancestry informative (AI) SNPs were identified as being fixed for different alleles in a sample of two pure *M. esculenta* (Albert and CM33064) and two pure *M. glaziovii*.

Out of 173,647 SNP in our imputed dataset, 12,502 matched published AI SNPs. For these AI SNPs, we divided each chromosome into non-overlapping windows of 20 SNP. Within each window, for each individual, we calculated the proportion of SNPs that were *M*. *glaziovii* homozygous (G/G), *M. esculenta* homozygous (E/E), or heterozygous (G/E). We assigned G/G, G/E or E/E ancestry to each window, for each individual only when the proportion of the most common genotype in that window was at least twice the proportion of the second most common genotype. If this was not the case, we assigned windows a “No Call” status.

### Linkage disequilibrium plots

To confirm the presence of a haplotype on chromosome 4, we calculated LD scores for every SNP marker on chromosome 4 in a 1 Mb window using GCTA^63^. Briefly, the LD score for a given marker is calculated as the sum of R^2^ adjusted between the index marker and all markers within a specified window:

5 $${R}_{adjusted}^{2}={R}^{2}-\frac{\,(1-\,{R}^{2})}{(n-2)}$$

where “n” is the population size and R^2^ is the usual estimator of the squared Pearson’s correlation^76^. The resulting LD scores were then plotted against log_10_(P-value) from GWAS of every marker on chromosome 4.

To highlight the importance of the associated markers on chromosome 11 we calculated pairwise squared Pearson’s correlation coefficient (*r*^2^) between the top significant GWAS SNP hit on this chromosome and neighboring markers in a window of 2 Mb (1 Mb upstream and 1 Mb downstream)^77,78^.

### Candidate gene identification

We used the *mlma* GCTA output to filter out SNP markers based on −log_10_(P-value) > 5.9. The resulting significant SNP markers were then mapped onto genes using the SNP location and gene description from the M.esculenta_305_v6.1.gene.gff3 available in Phytozome 11^58^ for *Manihot esculenta* v6.1 using bedtools^79^.

### Data availability

The phenotypic and genotypic data generated and analyzed during this study are available in the Cassavabase repository, https://www.cassavabase.org/.

## Electronic supplementary material

Supplementary Figures

Supplementary Tables and Data
