# Supplementary material for: Genome-wide association mapping and genomic prediction for CBSD resistance in Manihot esculenta
Source: Sci Rep. 2018 Jan 24;8:1549. doi: 10.1038/s41598-018-19696-1 (PMC5784162; doi:10.1038/s41598-018-19696-1)

Genome-wide association mapping and genomic prediction for CBSD resistance in *Manihot esculenta*

Siraj Ismail Kayondo[1,2*+], Dunia Pino Del Carpio[3+], Roberto Lozano[3], Alfred Ozimati[1,3], Marnin Wolfe[3], Yona Baguma[1], Vernon Gracen[2,3], Samuel Offei [2], Morag Ferguson[5], Robert Kawuki[1] and Jean-Luc Jannink[3,4]

[1] National Crop Resources Research Institute, NaCRRI, P.O. Box, 7084 Kampala, Uganda
[2] West Africa Center for Crop Improvement, (WACCI), University of Ghana, Ghana
[3] School of Integrative Plant Sciences, Section on Plant breeding and Genetics, Cornell University, Ithaca, New York, USA
[4] US Department of Agriculture – Agricultural Research Service (USDA-ARS), USA
[5] International Institute for Tropical Agriculture (IITA), Nairobi, Kenya

*Corresponding author: skayondo@wacci.edu.gh

+ These authors contributed equally to this work

Supplementary Figure S1. **Cassava brown streak disease symptoms on leaves and roots**. Symptom severity score ranges from 1 (no visible symptoms) to 5 (severely disease plants) on leaves (**A**) and storage roots (**B**).

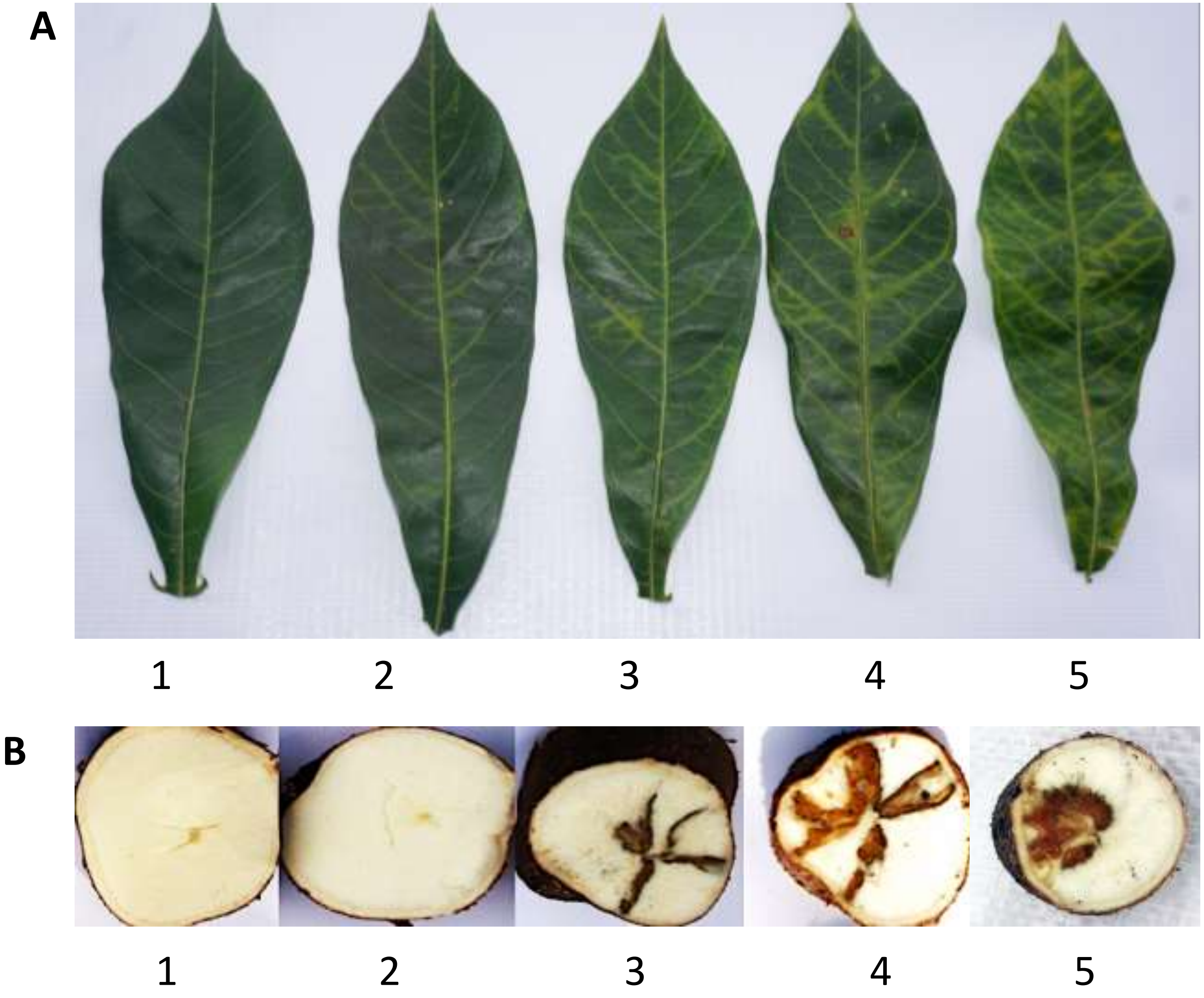

Supplementary Figure S2.Phenotypic distribution of CBSD severity traits in Panel 1.

(A) de-regressed BLUPs distribution of CBSD foliar severity at 3 MAP (B) de-regressed BLUPs distribution of CBSD foliar severity at 6MAP (C) de-regressed BLUPs distribution of CBSDRS 12 months root severity. MAP = months after planting. The de-regressed BLUP value is on x-axis and the number of clones is presented on the y-axis.

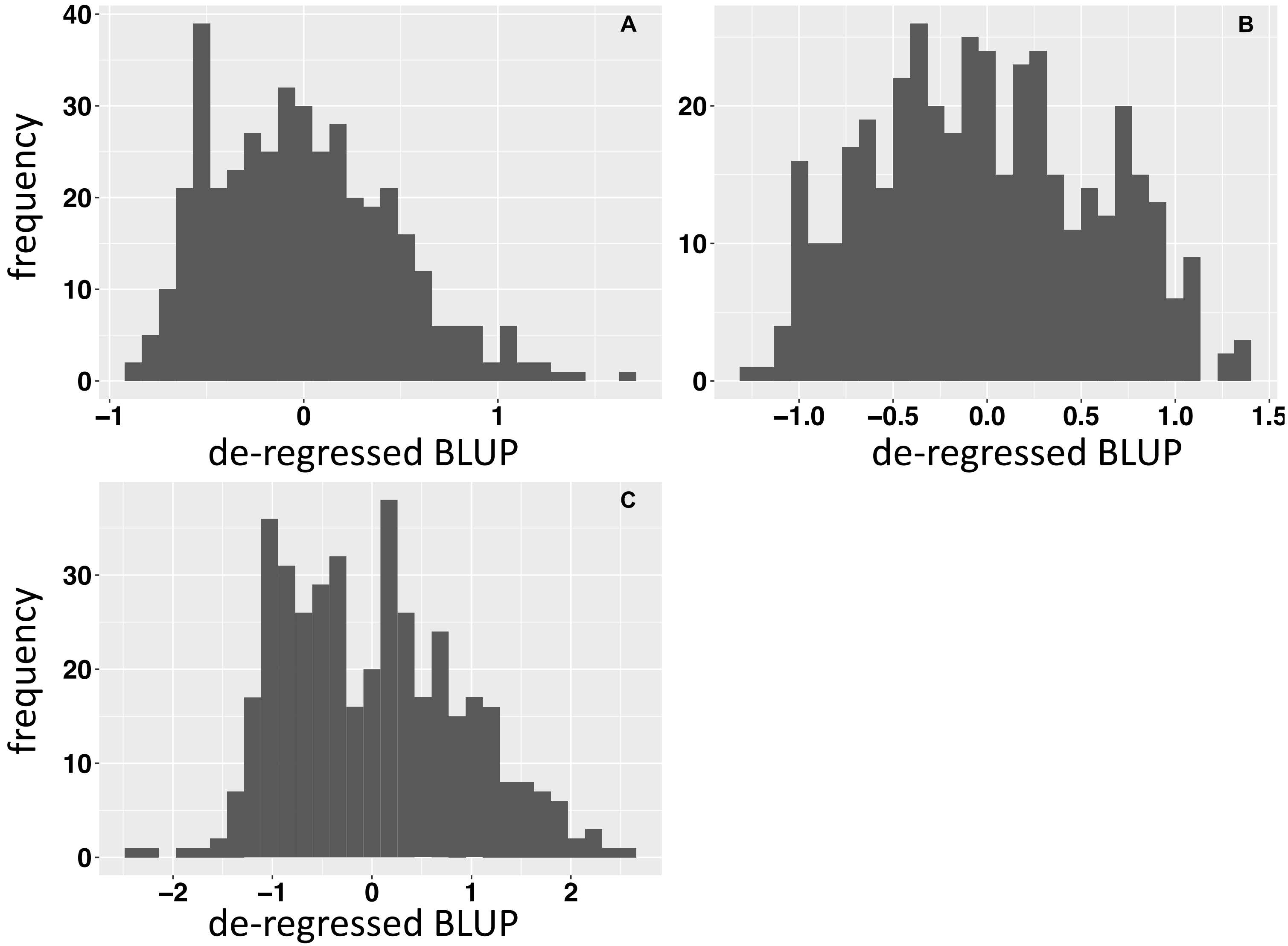

Supplementary Figure S3.Phenotypic distribution of CBSD severity traits in Panel 2.
(A) de-regressed BLUPs distribution of CBSD foliar severity 3 MAP (B) de-regressed BLUPs distribution of CBSD foliar severity 6MAP (C) de-regressed BLUPs distribution of CBSD foliar severity 9 MAP(D) de-regressed BLUPs distribution of CBSDRS 12 months root severity. MAP = months after planting. The de-regressed BLUP value is on x-axis and the number of clones is presented on the y-axis.

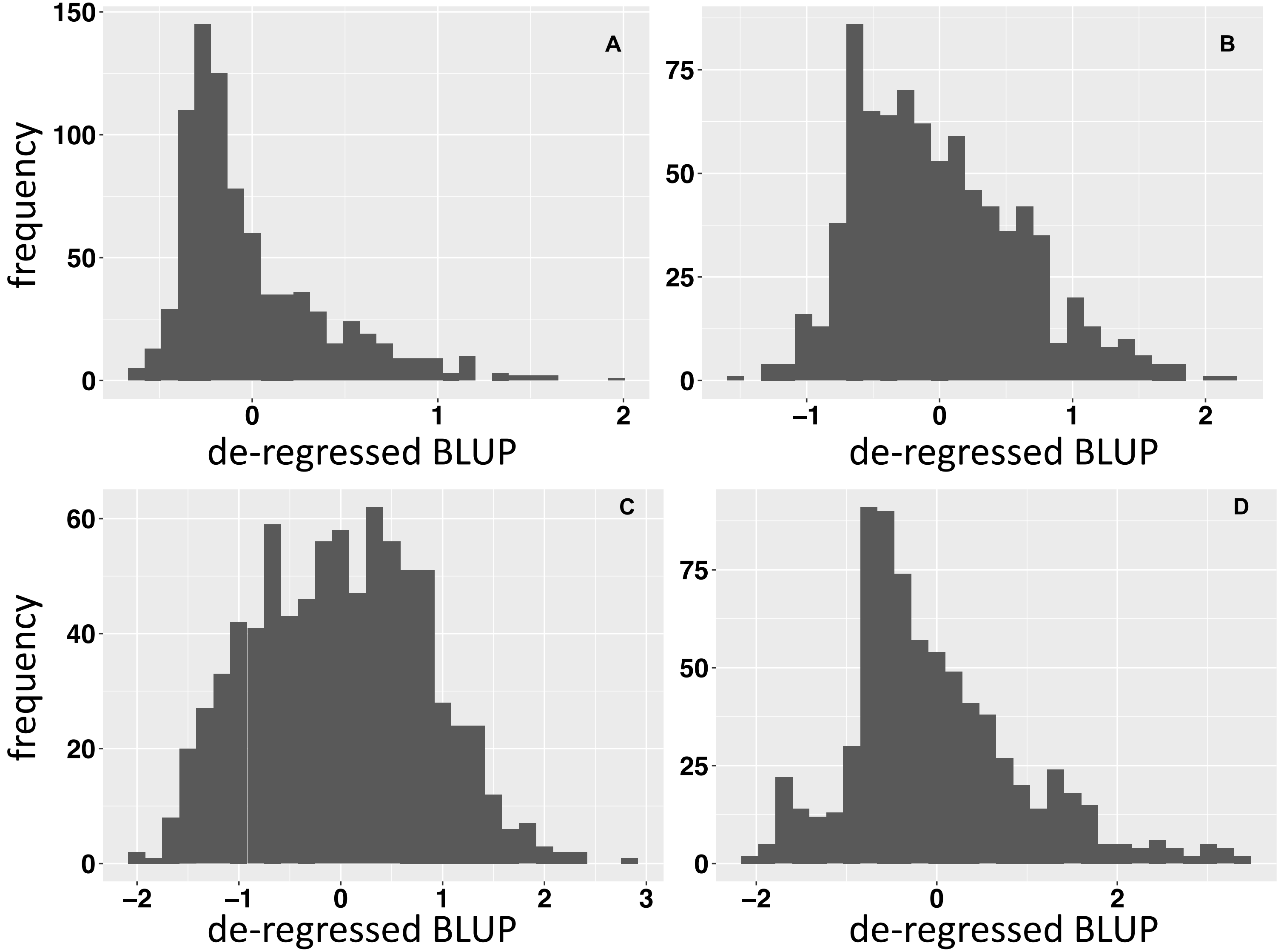

Supplementary Figure S4. Correlation plots between de-regressed BLUPs for foliar and root symptoms. Pairwise correlation plots of de-regressed BLUPs (x and y axes) measured in panel 1 at Namulonge (Nam), Ngetta (Nge) and Kasese (Kas) and in panel 2 at Nam, Kamuli (Kam) and Serere (Ser). De-regressed BLUPs were computed from CBSD symptom severity scores measured at 3 MAP ,6MAP , 9MAP and 12 months at harvest time (CBSDRS=root severity).MAP = months after planting, drBLUP = de-regressed BLUP.

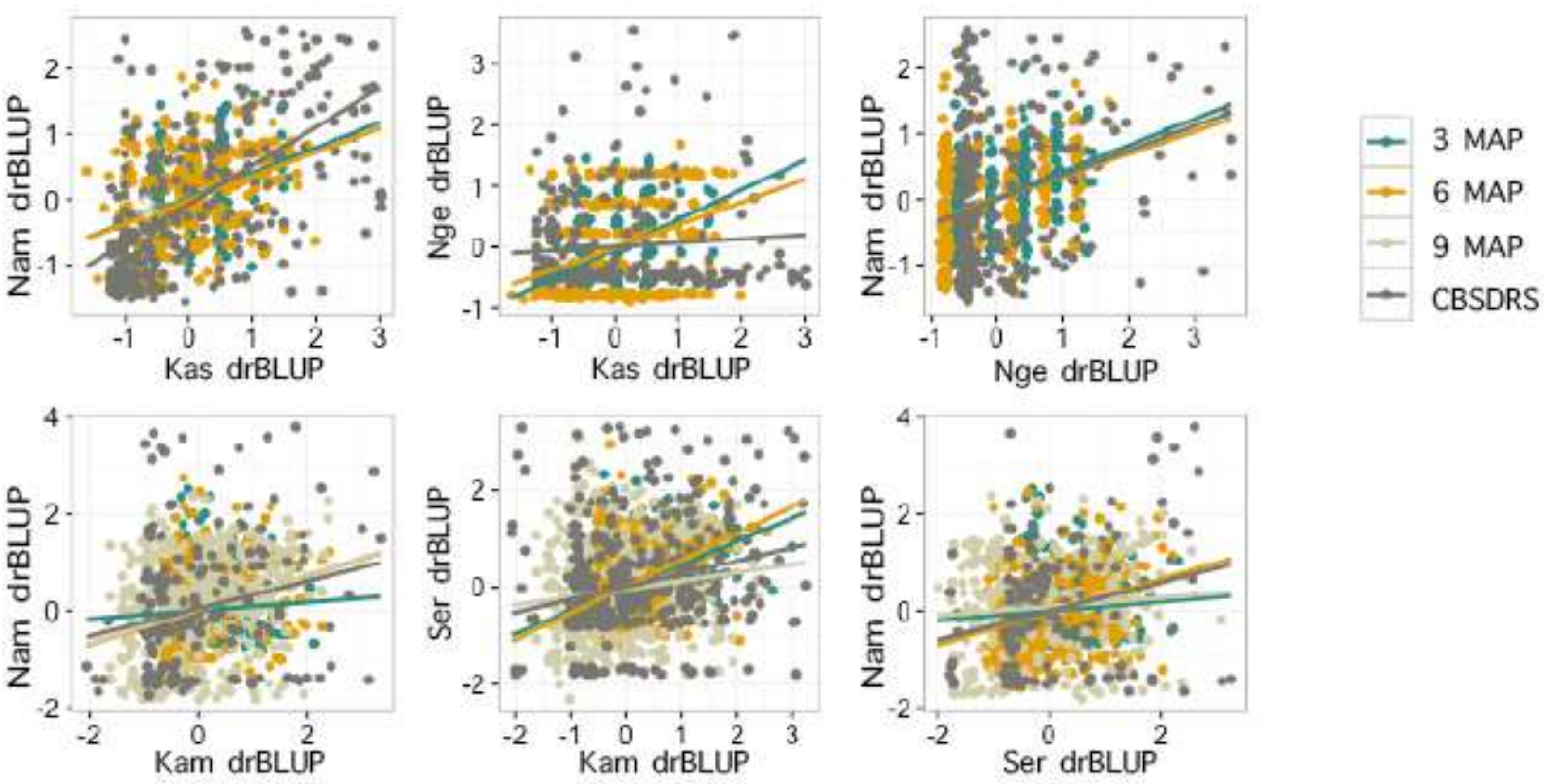

Supplementary Figure S5: Manhattan plots for CBSD severity traits in Kasese – Panel1

Association tests were performed for CBSD symptom severity on leaves at 3 and 6 month after planting (MAP) and on roots 12 MAP (CBSDRS). The horizontal line indicates the genome-wide significance level ($-\log_{10}$ (P-value) = 5.9).

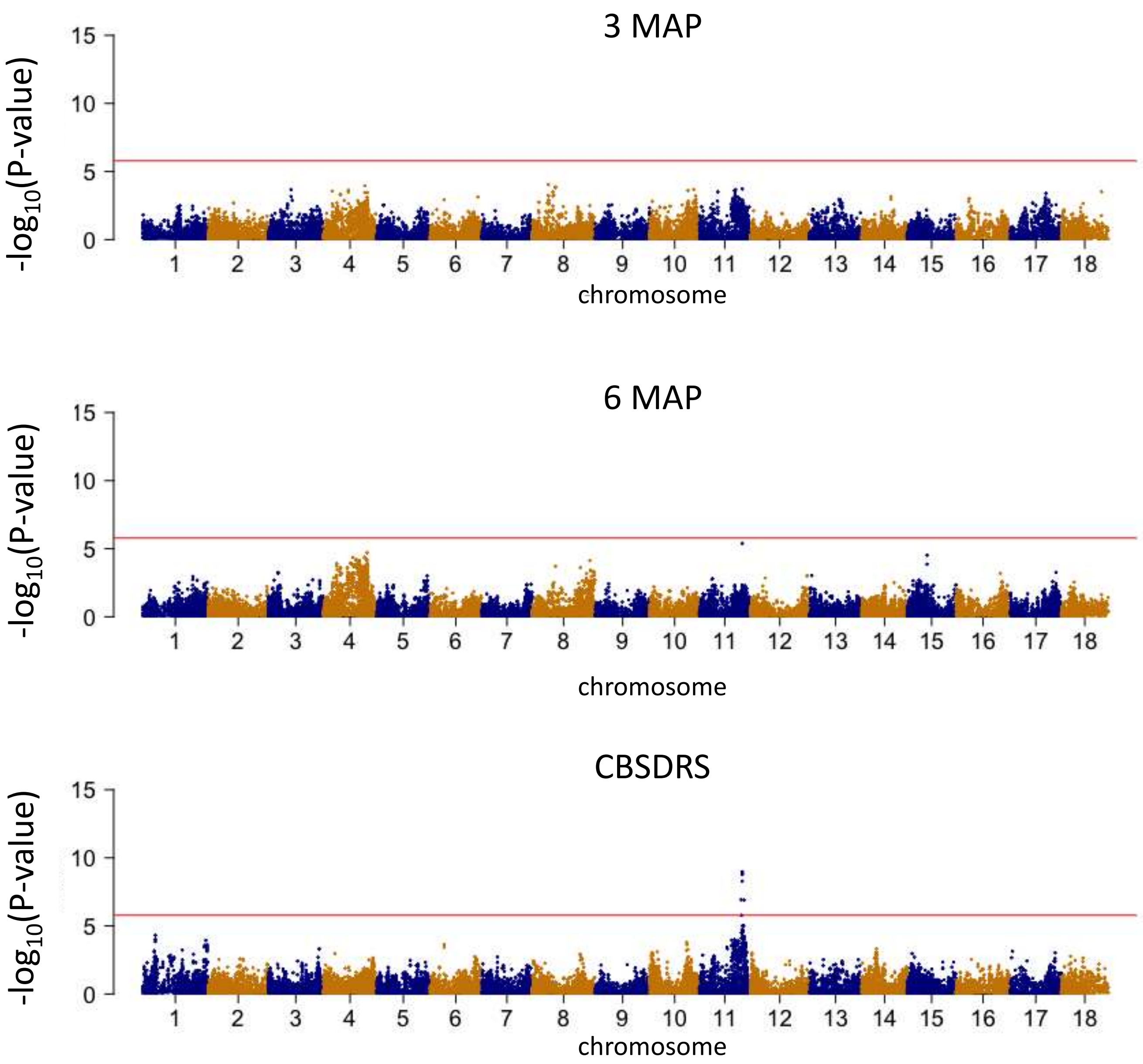

Supplementary Figure S6: Manhattan plots for CBSD severity traits in Ngetta – Panel1.
Association tests were performed for CBSD symptom severity on leaves at 3 and 6 month after planting (MAP) and on roots 12 MAP (CBSDRS). The horizontal line indicates the genome-wide significance level ($-\log_{10}$ (P-value) = 5.9).

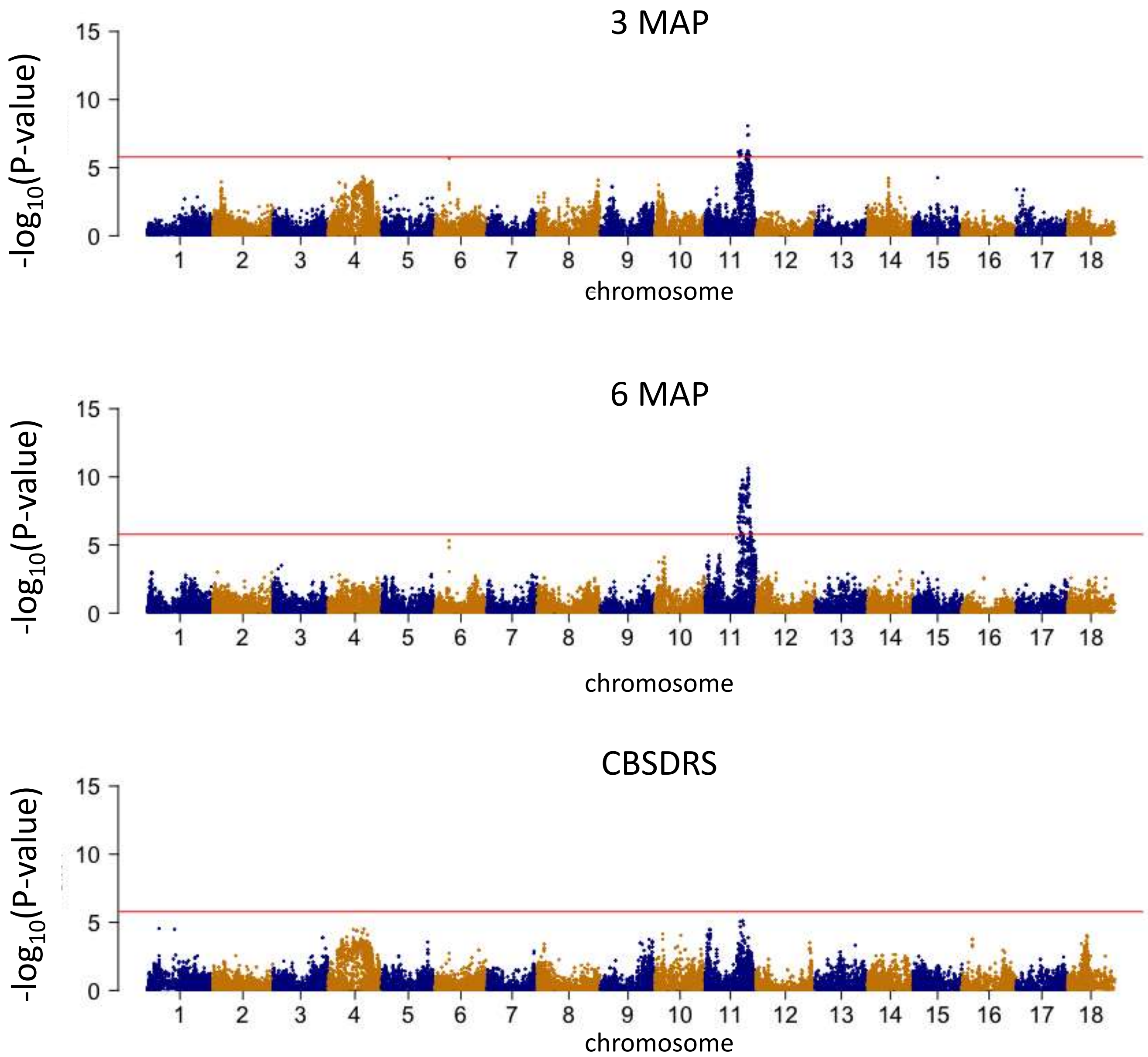

Supplementary Figure S7: Manhattan plots for CBSD severity traits in Namulonge – Panel1. Association tests were performed for CBSD symptom severity on leaves at 3 and 6 month after planting (MAP) and on roots 12 MAP (CBSDRS). The horizontal line indicates the genome-wide significance level ($-\log_{10}$ (P-value) = 5.9).

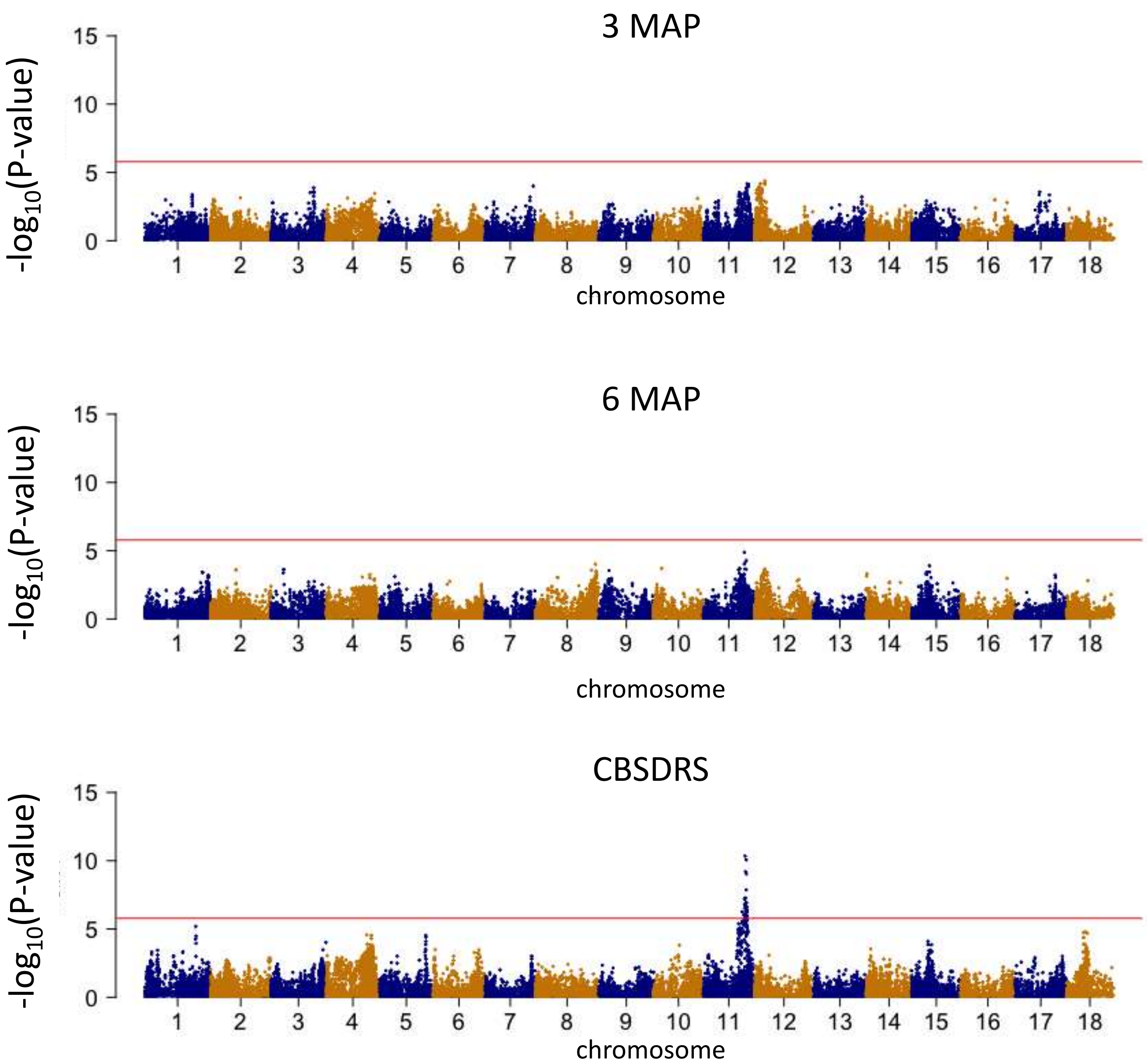

Supplementary Figure S8: Manhattan plots for CBSD severity traits for Panel1 – Multi-locational dataset. Association tests were performed for CBSD symptom severity on leaves at 3 and 6 month after planting (MAP) and on roots 12 MAP (CBSDRS). The horizontal line indicates the genome-wide significance level ($-\log_{10}$ (P-value) = 5.9).

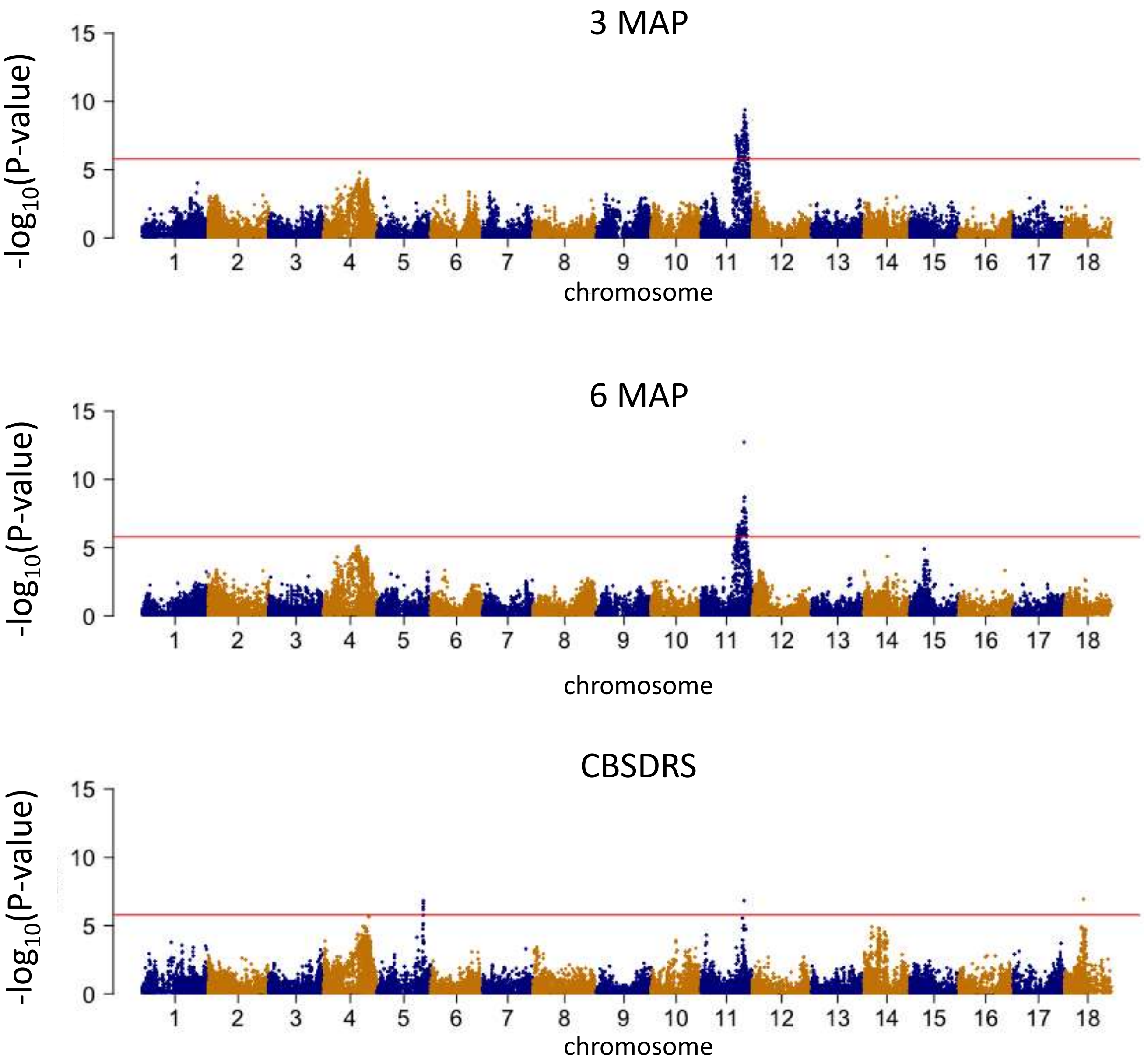

Supplementary Figure S9: Manhattan plots for CBSD severity traits in Kamuli – Panel2. Association tests were performed for CBSD symptom severity on leaves at 3 ,6 and 9 month after planting (MAP), and on roots 12 MAP (CBSDRS). The horizontal line indicates the genome-wide significance level ($-\log_{10}$ (P-value) = 5.9).

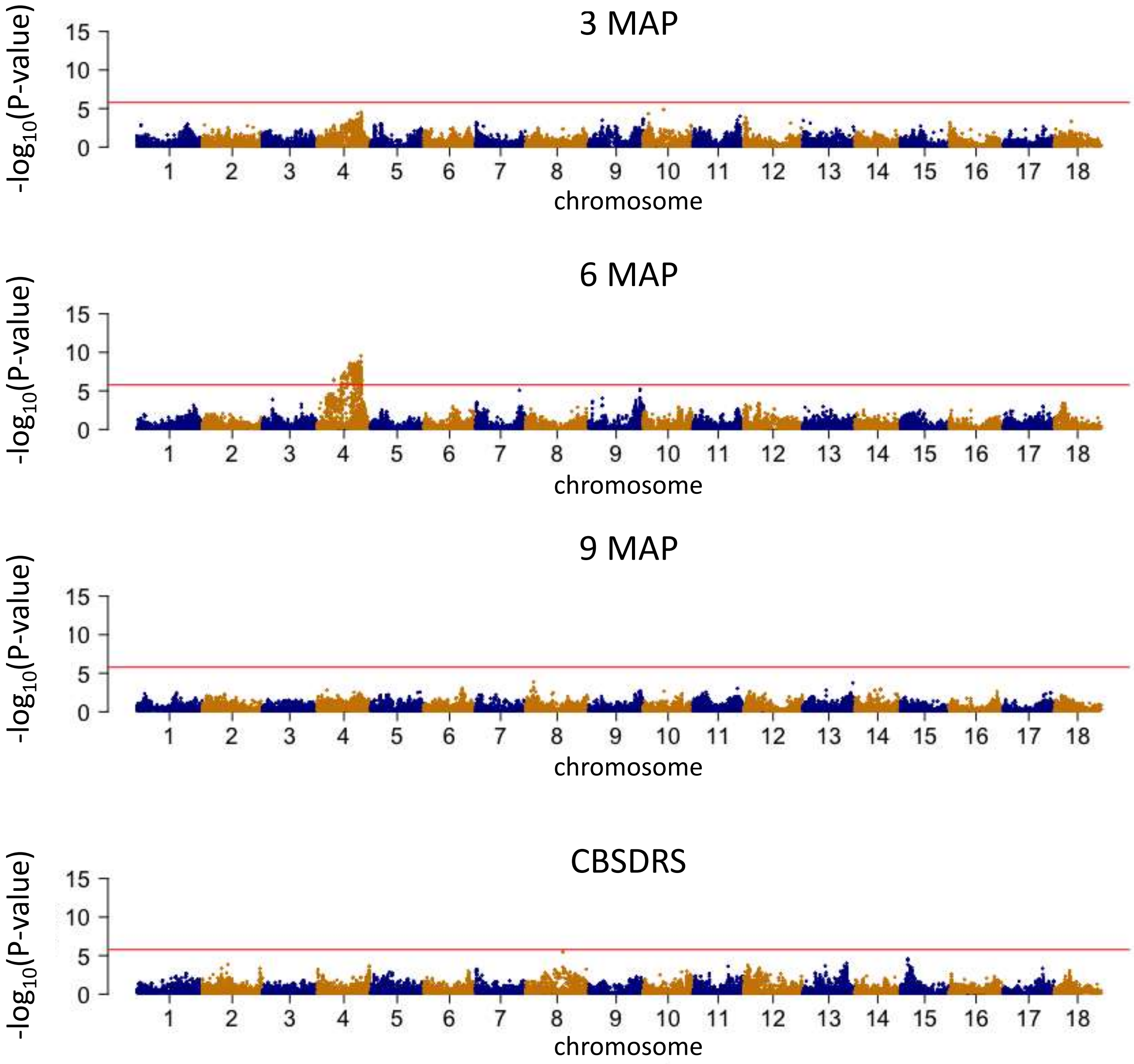

Supplementary Figure S10: Manhattan plots for CBSD severity traits in Namulonge – Panel2.
Association tests were performed for CBSD symptom severity on leaves at 3 ,6 and 9 month after planting (MAP), and on roots 12 MAP (CBSDRS). The horizontal line indicates the genome-wide significance level ($-\log_{10}$(P-value) = 5.9).

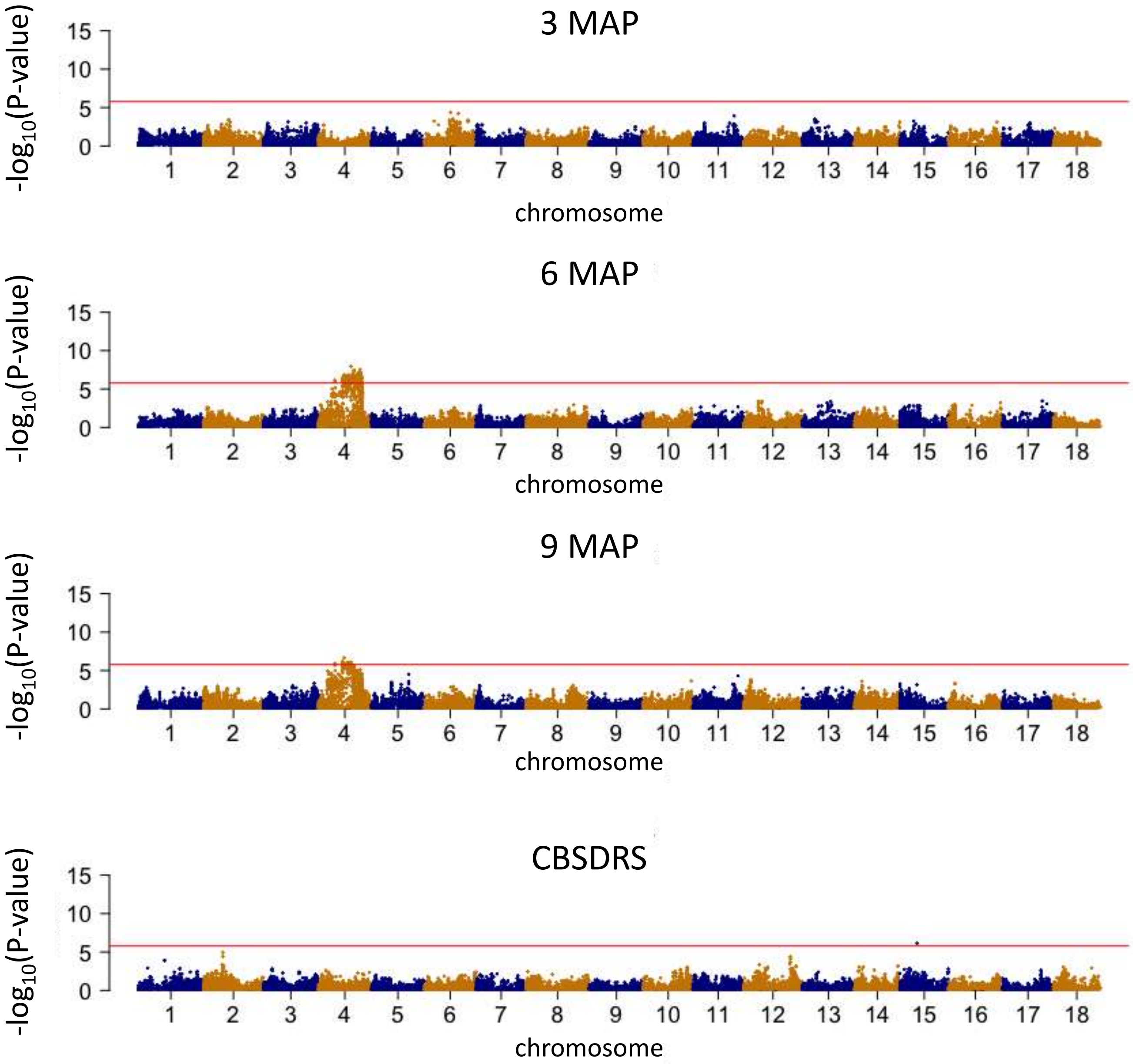

Supplementary Figure S11: Manhattan plots for CBSD severity traits in Serere – Panel2.
Association tests were performed for CBSD symptom severity on leaves at 3 ,6 and 9 month after planting (MAP), and on roots 12 MAP (CBSDRS). The horizontal line indicates the genome-wide significance level ($-\log_{10}$(P-value) = 5.9).

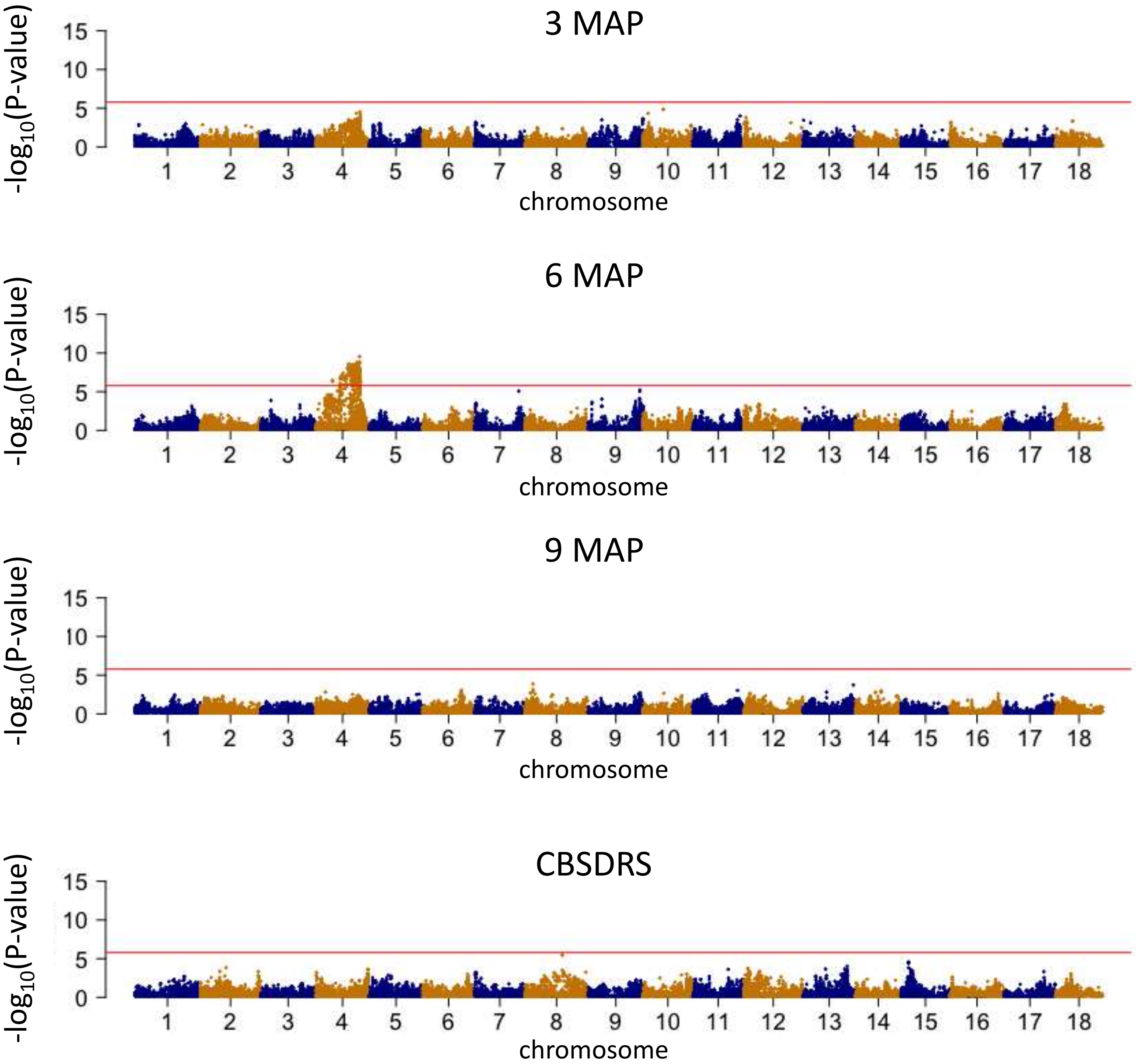

Supplementary Figure 12: Manhattan plots for CBSD severity traits for Panel2 – Multi-location.
Association tests were performed for CBSD symptom severity on leaves at 3 ,6 and 9 month after planting (MAP), and on roots 12 MAP (CBSDRS). The horizontal line indicates the genome-wide significance level ($-\log_{10}$(P-value) = 5.9).

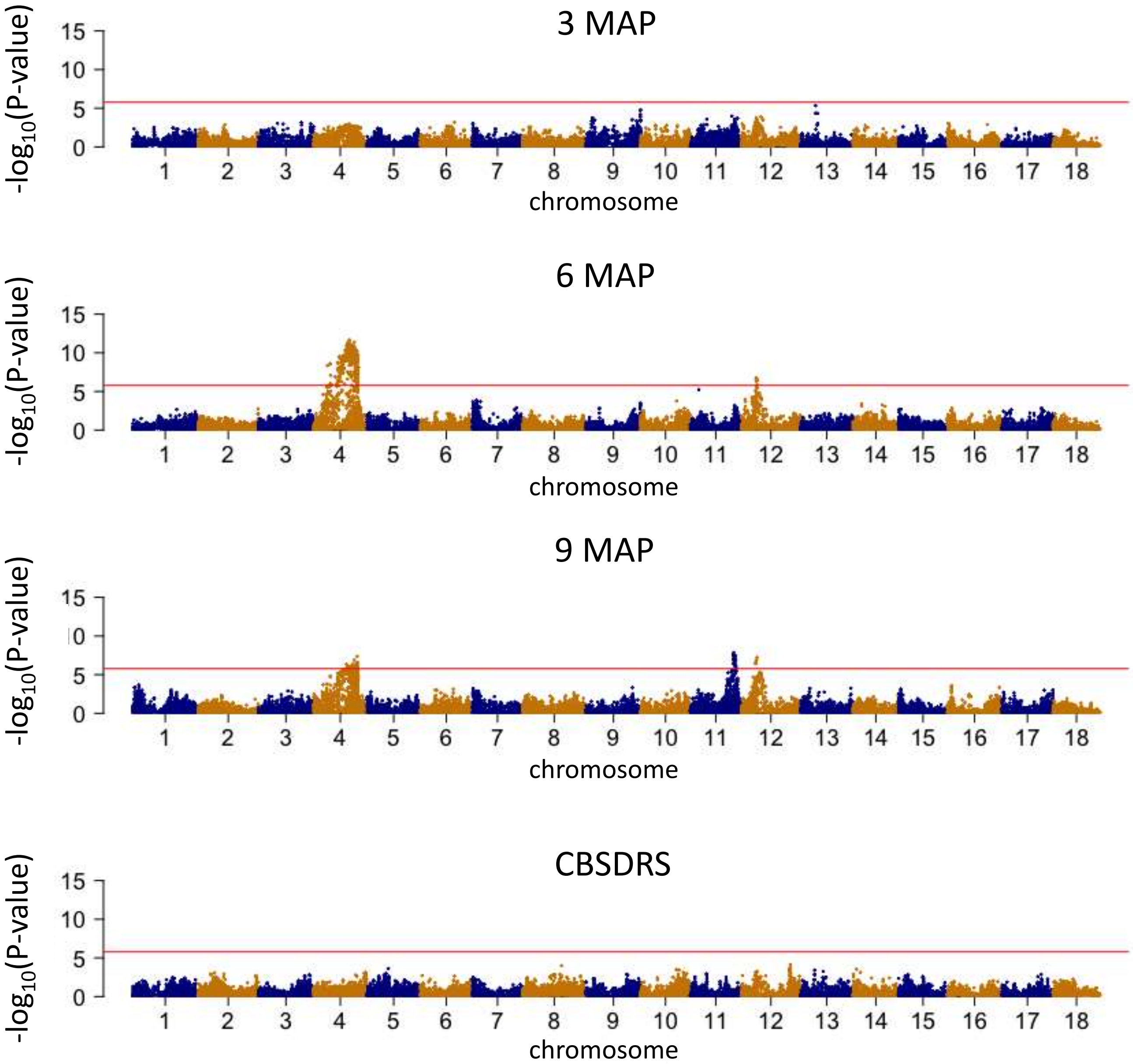

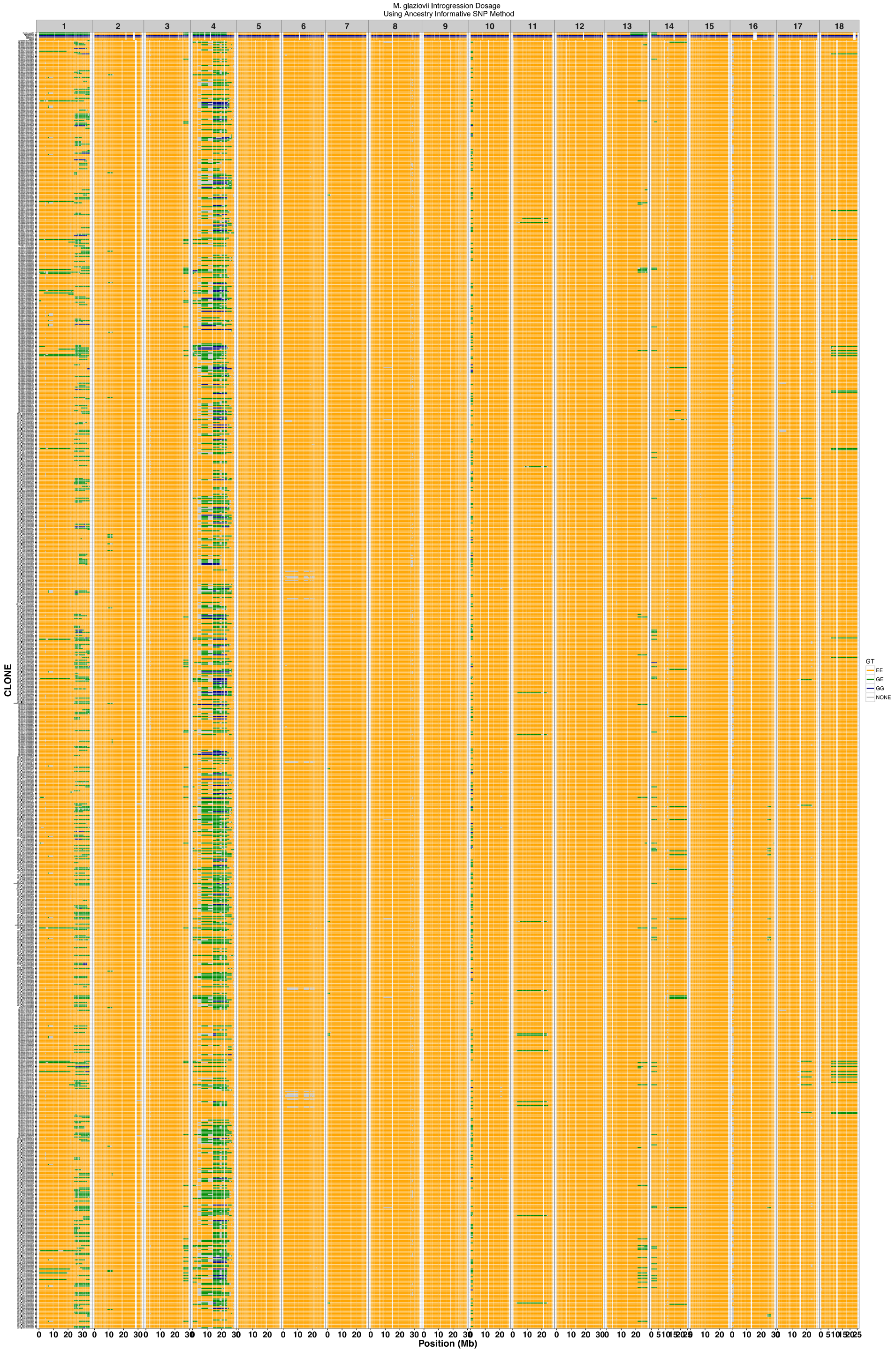

Supplementary figure 13.Combined panels dataset analysis of introgression Regions using the ancestry informative method. For each clone of the two GWAS panels we calculated the proportion of genotypes that were homozygous (G/G, blue) or heterozygous (G/E, green) for *M. glaziovii* allele and the proportion that were homozygous for the *M. esculenta* allele (E/E, orange).

Supplementary Figure S14. GWAS results for 6MAP CBSD severity combined panels multilocational dataset.**(a)** GWAS results for 6MAP CBSD symptom severity without CMD correction **(b)** GWAS results using CMD scoring correction in the calculation of the de-regressed BLUPs.

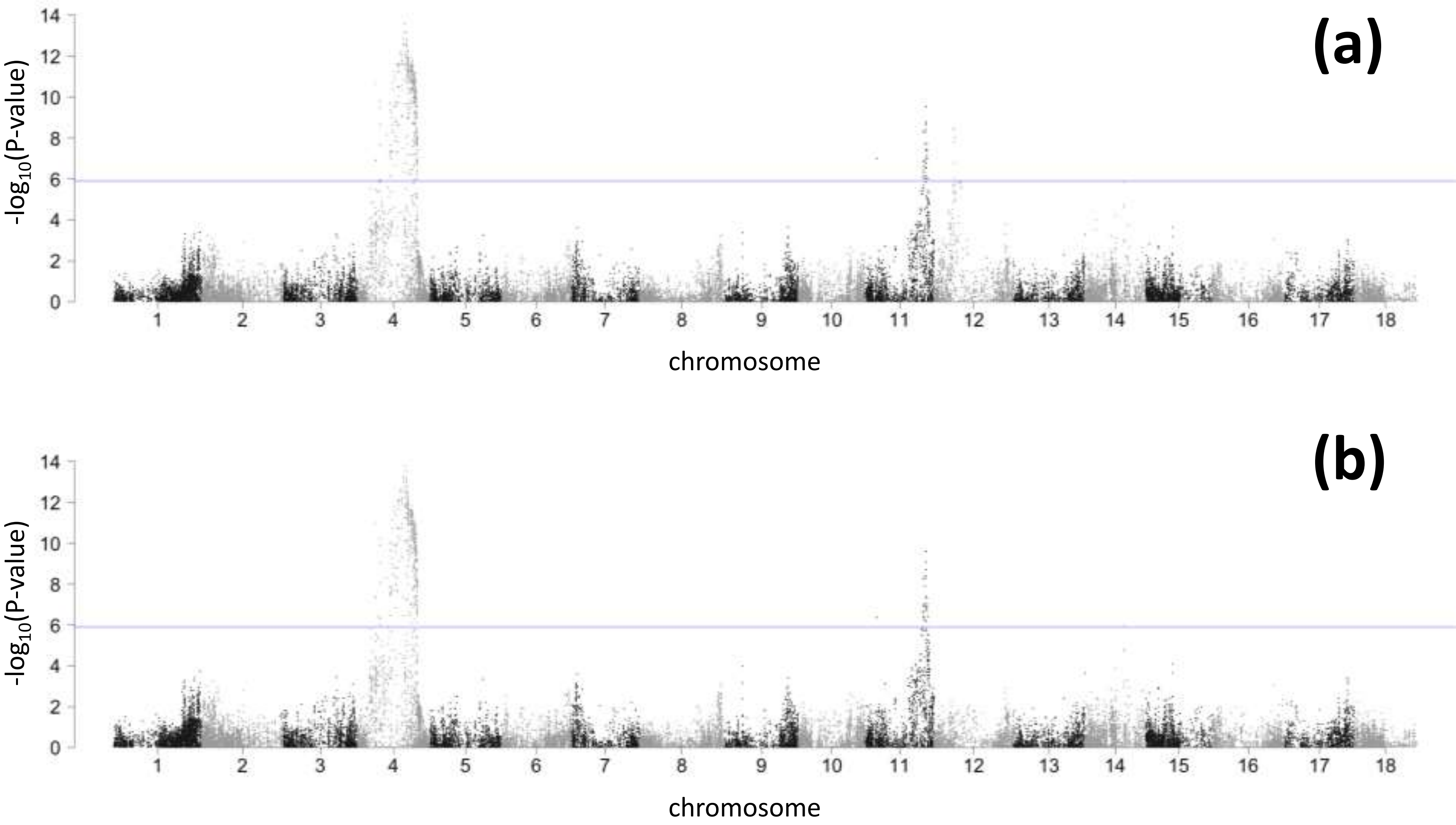

Supplementary Figure S15. Cross validation accuracies of the Multi-kernel GBLUP approach compared to the single kernel GBLUP. GS predictive accuracies results for CBSD severity were calculated using the combined panels multilocational dataset. Cross validation results are shown for CBSD 3 MAP(CBSD3S),CBSD 6 MAP (CBSD6S) and root necrosis severity (CBSDRS).MAP= months after planting. Cross validation accuracies per kernel are presented : Chr 4 = chromosome 4, Chr 11 = chromosome 11 and RestGeno= other chromosomes (minus 4 and 11). TotalAcc = total predictive accuracy of the multikernel GBLUP, GBLUP= single kernel GBLUP.

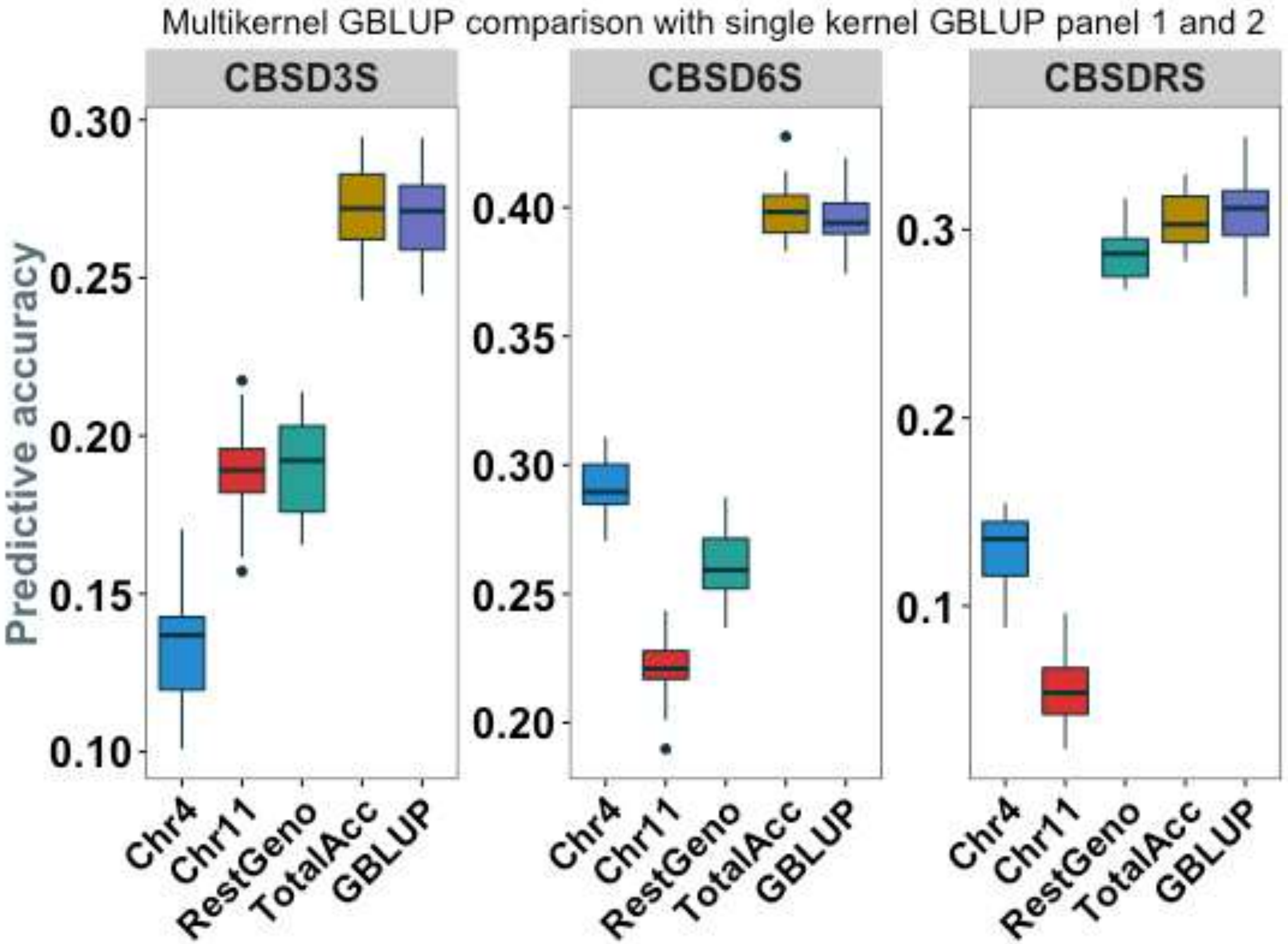

Supplement: Supplementary file 1 — Supplementary Figures [file 41598_2018_19696_MOESM1_ESM.pdf]
